# KPNA1 regulates nuclear import of NCOR2 splice variant BQ323636.1 to confer tamoxifen resistance in breast cancer

**DOI:** 10.1002/ctm2.554

**Published:** 2021-10-12

**Authors:** Ho Tsoi, Ellen PS Man, Man‐Hong Leung, Ka‐Chun Mok, Ka‐Man Chau, Lai‐San Wong, Wing‐Lok Chan, Sum‐Yin Chan, Mai‐Yee Luk, Cheuk‐Nam Cheng, Ui‐Soon Khoo

**Affiliations:** ^1^ Department of Pathology LKS Faculty of Medicine The University of Hong Kong, Hong Kong SAR China; ^2^ Department of Clinical Oncology Queen Mary Hospital Hong Kong China; ^3^ Department of Clinical Oncology LKS Faculty of Medicine The University of Hong Kong China

Dear Editor,

Tamoxifen is a first‐line treatment option for estrogen‐receptor‐α positive (ER+) breast cancer. Drug resistance significantly compromises its clinical efficacy. Nuclear receptor corepressor‐2 (NCOR2) is a transcriptional coregulatory protein. We previously identified a novel splice variant of NCOR2, that is, BQ323636.1 (BQ), which retains only the N‐terminus repression domain‐1 of the NCOR2 wild‐type protein (Figure S1).^1^ BQ nuclear overexpression is found significantly associated with tamoxifen resistance in ER+ primary breast cancer, nuclear localization being essential in modulating tamoxifen response.^2^ This study reports a possible molecular mechanism behind BQ nuclear localization mediated by KPNA1 (importin‐α5).

We generated two expression constructs in which the BQ expression vector was fused with either a nuclear‐localization signal (BQ‐NLS) or with a nuclear‐export signal (BQ‐NES), and confirmed that BQ‐NLS was predominantly localized in the nucleus, further promoted cell proliferation and enhanced tamoxifen resistance (Figure S2A–D). Using cNLS Mapper,^3^ we identified a putative NLS (PQRRRPSLLS) in BQ (NLS_BQ_; Figure S3A). Through RaptorX,^4^ we found that the NLS in BQ had greater relative surface accessibility than for that in NCOR2 (Figure S3B), suggesting it might be more functional. By coimmunoprecipitation, only KPNA1 interacted with BQ and importin‐β1 (Figure 1A). An expression construct that expressed GFP fused with NLS_BQ_ was cloned and coimmunoprecipitation confirmed that GFP‐NLS_BQ_ could interact with KPNA1 (Figure S3C). Knockdown of KPNA1 resulted in reduced nuclear‐BQ (Figures 1B, C and S4A–C) in BQ‐overexpressed cells. LCC2, a tamoxifen resistant cell‐line derived from MCF‐7, has a high endogenous BQ‐expression (Figure S5A). Knockdown of KPNA1 in LCC2 reduced BQ levels in the nucleus (Figures 1D and S5B–D). These results suggested that NLS_BQ_ was functional and KPNA1 may mediate the nuclear import of BQ in breast cancer cells.

**FIGURE 1:**
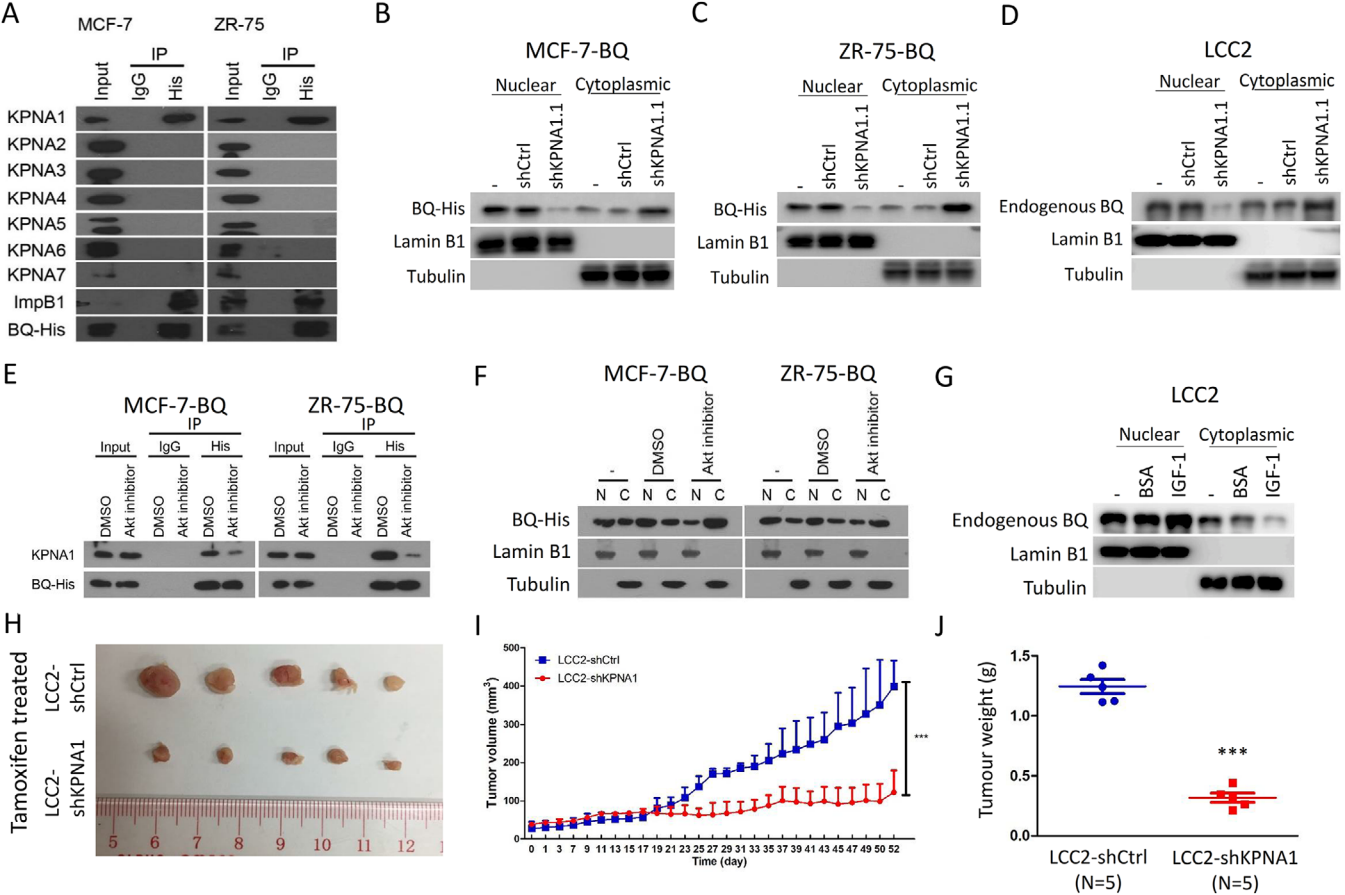
KPNA1 modulated the nuclear import of BQ and thus the response to tamoxifen in breast cancer. (A) KPNA1 was found to interact with BQ in MCF‐7 and ZR‐75. The cells were stably transfected with pcDNA3.1‐His‐BQ. Immunoprecipitation with anti‐His tag was performed. Western blot was employed to determine the presence of the indicated protein candidate in the immunoprecipitant. Knockdown of KPNA1 could alter the subcellular localization of BQ in both stable BQ overexpressing cells (B) MCF‐7‐BQ and (C) ZR‐75‐BQ. The nontargeting shRNA (shCtrl) and KPNA1 targeting shRNA (shKPNA1.1) was used for transfection. Nucleocytoplasmic fractionation was performed after 48 h posttransfection. (D) Knockdown of KPNA1 could reduce the level of BQ in the nuclear fraction of LCC2. shKNPA1.1 was employed to downregulate the expression of KPNA1. Western blot was employed to determine levels of the indicated protein candidates. Lamin B1 and tubulin was used as the nuclear and cytoplasmic markers respectively. (E) Inhibition of AKT could compromise the interaction between KPNA1 and BQ. Stable BQ overexpression cells MCF‐7‐BQ and ZR‐75‐BQ were used. 1 μM of the AKT inhibitor was used for treating the cells for 72 h. Immunoprecipitation with anti‐His was performed. (F) Inhibition of AKT could alter the subcellular localization of BQ in MCF‐7‐BQ and ZR‐75‐BQ cells. 1 μM of the AKT inhibitor was used for treating the cells for 72 h. (G) IGF‐1 treatment could enhance the level of BQ in nuclear fraction. LCC2 cells were treated with 10 nM of IGF‐1 for 24 h. Nucleocytoplasmic fractionation was performed. Western blot was employed to determine the level of the indicated protein candidates. Lamin B1 and tubulin was used as the nuclear and cytoplasmic markers respectively. All experiments were repeated three times. (H) Downregulation of KPNA1 could recover tamoxifen sensitivity in vivo. Xenografts were established from LCC2‐shCtrl (*N* = 5) and LCC2‐shKPNA1 (*N* = 5) cell lines in mammary fat‐pads of nude mice. shCtrl and shKPNA1.1 were employed to established the stable cell lines LCC2‐shCtrl and LCC2‐shKPNA1. Tamoxifen (0.5 mg/mouse) was used to treat the mice in both of the groups twice per week. The treatment started at day 7. Tumors were isolated and shown in the photo. (H) Statistical analysis of (I). Results were shown as mean ± SD from 5 mice. Student's *t*‐test was employed to determine the statistical significance. (J) Knockdown of KPNA1 could significantly reduce tumor weight. Each spot represents one of the tumors. Student's *t*‐test was employed. ***p < .001

Using GPS tool,^5^ it was predicted that serine in NLS_BQ_ could be phosphorylated by AKT (Figure S6A). Constructs were created expressing GFP fused with wild‐type NLS_BQ_ (wtNLS_BQ_) and with mutant NLS_BQ_ (mtNLS_BQ_; Figure S6B). Compared with wtNLS_BQ_, coimmunoprecipitation showed that the interaction between KPNA1 and mtNLS_BQ_ was significantly compromised (Figure S6C). Furthermore, AKT inhibitor treatment reduced the interaction between KPNA1 and wtNLS_BQ_ (Figure S6D) as well as the interaction between KPNA1 and BQ (Figure 1E). AKT inhibition compromised the nuclear import of BQ (Figure 1F). To validate that nuclear import of BQ can be modulated by AKT, we employed IGF‐1 to activate AKT activity (Figure S6E) in endogenously BQ‐overexpression cells LCC2 and found IGF‐1 could enrich BQ levels in the nucleus (Figure 1G). These results suggest that AKT is involved in governing the subcellular localization of BQ in breast cancer via KPNA1.

Knockdown of KPNA1 could recover tamoxifen sensitivity in vitro (Figure S7A–H). While KPNA5 and KPNA6 showed high similarity to KPNA1 (Figure S8A), knockdown of either did not alter tamoxifen resistance in LCC2 (Figure S8B–D), suggesting KPNA1 to be specific for mediating tamoxifen resistance. In vivo studies showed KPNA1 knockdown xenografts could recover tamoxifen response (Figure 1H–J). Therefore, knockdown of KPNA1 compromises the effect of high BQ‐expression in conferring tamoxifen resistance.

From our previous informatics study, we observed HIF‐1α signaling pathway enrichment in BQ‐overexpressing cells^6^ which may contribute to tamoxifen resistance.^7^ We confirmed that BQ‐overexpression could enhance both mRNA (Figure 2A) and protein expression (Figure 2B) of HIF‐1α under normal and hypoxic conditions. As expected, BQ‐overexpression enhanced HIF‐1α transcriptional activity as indicated by luciferase reporter assay (Figure 2C) and qPCR (Figure 2D) as revealed by the expression of HIF‐1α‐regulated genes, hexokinase (HK), phosphofructokinase‐1 (PFK), enolase‐1 (ENO1), and lactate dehydrogenase‐A (LDHA).^8^ Moreover, knockdown of KPNA1 (Figure S9A–C) compromised the effect of BQ on HIF‐1α expression (Figure 2E) and the activity of HIF‐1α (Figure 2F). Similar results were obtained from LCC2 (Figure S10A, B). These results suggest that nuclear import of BQ should be important for the activity of HIF‐1α.

**FIGURE 2:**
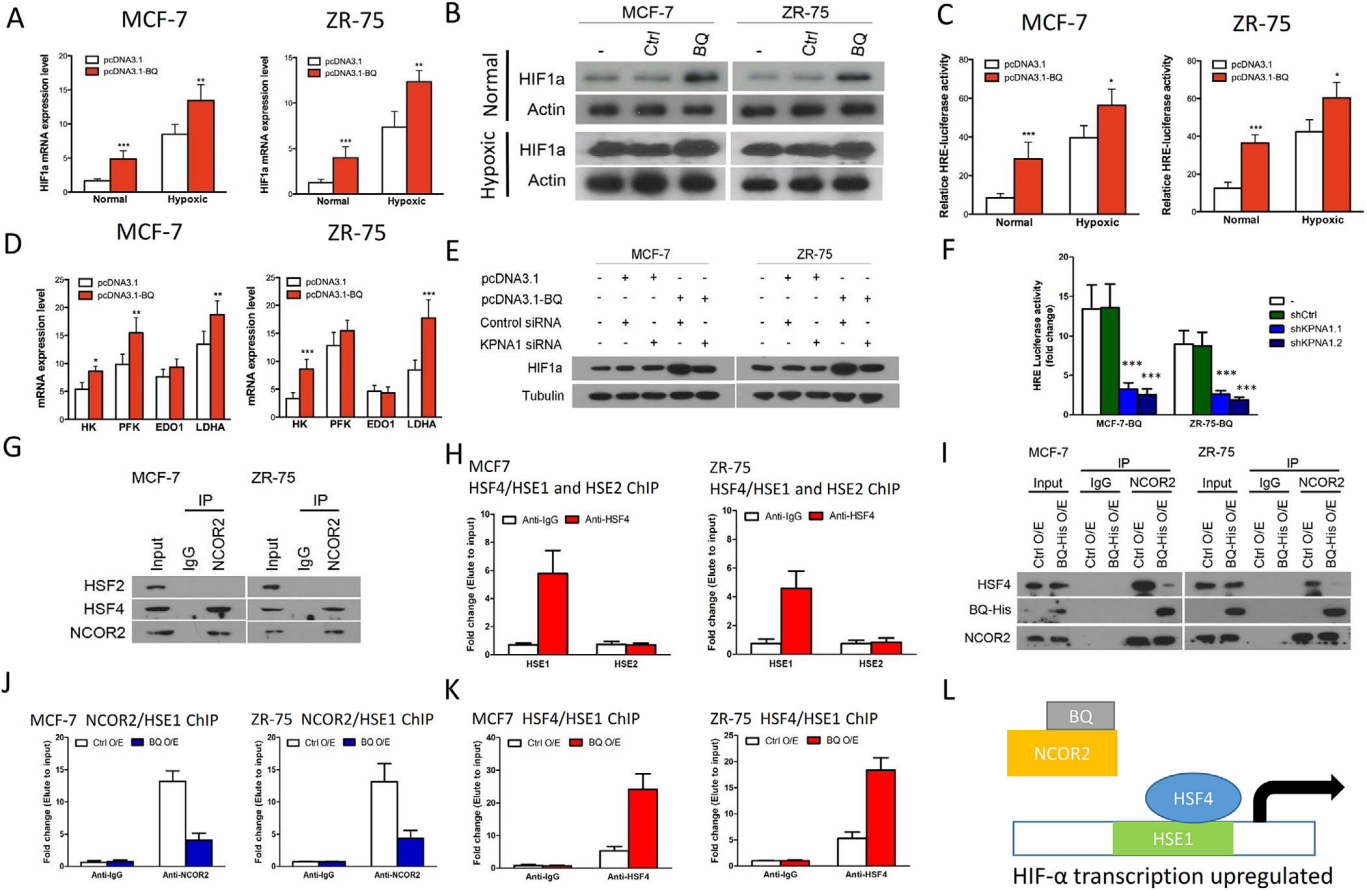
The molecular mechanism mediated by BQ to enhance the expression and activity of HIF‐1α in breast cancer. (A) Overexpression of BQ could enhance mRNA expression of HIF‐1α in both normal and hypoxic conditions. qPCR was performed. Actin was used as the internal control. Results were shown as mean ± SD from three independent experiments. Student's *t*‐test was employed to compare the statistical significance with the control. (B) Overexpression of BQ could enhance protein expression of HIF‐1α in both normal and hypoxic conditions. Western blot was performed. Actin was used as the loading control. Representative images were shown. (C) Overexpression of BQ could enhance the transcription activity of HIF‐1α. Luciferase reporter assay with HIF‐1α response element (HRE) was employed. The cells were transiently transfected with HRE‐Luciferase reporter. The luciferase activity was determined after 48 h posttransfection. Results were shown as mean ± SD from three independent experiments. Student's *t*‐test was employed to determine the statistical significance compared with the control. (D) Overexpression of BQ could enhance the expression of HIF‐1α downstream target genes in nonhypoxic conditions. qPCR was performed. Actin was used as the internal control. Results were shown as mean ± SD from three independent experiments. Student's *t*‐test was employed to determine the statistical significance compared with the control. (E) Knockdown of KPNA1 could compromise the effect of BQ overexpression on HIF‐1α protein expression. The cells were transiently transfected with 0.5 μg of pcDNA3.1 or pcDNA3.1‐His‐BQ together with 20 pmol of nontargeting siRNA (control siRNA) or KPNA1 siRNA. Western blot was performed. Tubulin was used as the loading control. Representative images were shown. (F) Knockdown of KPNA1 could reduce the transcription activity of HIF‐1α. Stable cell lines were employed. The cells were transiently transfected with HRE‐Luciferase reporter. The luciferase activity was determined after 48 h posttransfection. Results were shown as mean ± SD from three independent experiments. Student's *t*‐test was employed to determine the statistical significance compared with untransfected control (–). (G) NCOR2 could interact with HSF4 in both MCF‐7 and ZR‐75. Immunoprecipitation with anti‐NCOR2 was performed. Western blot was employed to determine the presence of HSF2 and HSF4 in the immunoprecipitant. (H) HSF4 was found to interact with HSE1 but not with HSE2 in MCF‐7 and ZR‐75. ChIP assay was performed to determine the interaction between HSF4 and HSE region 1 (HSE1) and HSE region 2 (HSE2) within the promoter region of HIF‐1α. qPCR was employed to determine the relative amount of HSE1 and HSE2 amplicon in the elutant. Results were shown as mean ± SD from three independent experiments. (I) Overexpression of BQ could compromise the physical interaction between NCOR2 and HSF4. Co‐IP was performed. NCOR2 was immunoprecipitated. Western blot was used to detect the presence of HSF4 and BQ in the immunoprecipitant. (J) Overexpression of BQ could compromise the interaction between NCOR2 and HSE1 in MCF‐7 and ZR‐75. Stable transfected cell lines MCF‐7‐Ctrl /ZR‐75‐Ctrl (empty pcDNA3.1) and MCF‐7‐BQ /ZR‐75‐BQ (pcDNA3.1‐His‐BQ) were used. ChIP assay was performed to determine the interaction between NCOR2 and HSE1. Immunoprecipitation with anti‐NCOR2 was performed. qPCR was performed to determine the relative amount of HSE1. Results were shown as mean ± SD from three independent experiments. Student's *t*‐test was employed to determine the statistical significance. (K) Overexpression of BQ could favour the interaction between HSF4 and HSE1 in MCF‐7 and ZR‐75. Stable transfected cell lines MCF‐7‐Ctrl /ZR‐75‐Ctrl (empty pcDNA3.1) and MCF‐7‐BQ/ZR‐75‐BQ (pcDNA3.1‐His‐BQ) were used. ChIP assay was performed to determine the interaction between HSF4 and HSE1. Immunoprecipitation with anti‐HSF4 was performed. qPCR was performed to determine the relative amount of HSE1. Results were shown as mean ± SD from three independent experiments. Student's *t*‐test was employed to determine the statistical significance. (L) Schematic diagram shows the proposed mechanism how BQ interferes with the interaction between NCOR2, HSF4 and HSE1 which in turn can alter the transcription of HIF‐1α

Heat shock factors HSF2 and HSF4 govern the transcription of HIF‐1α.^9^ Through coimmunoprecipitation, we found NCOR2 could interact with HSF4 (Figure 2G). There are two HSF4 binding sites in the promoter of HIF‐1α (Figure S11), namely HSE1 (–901 to –864) and HSE2 (–1457 to –1423). ChIP assay showed that HSF4 could bind to HSE1 but not to HSE2 (Figure 2H). BQ‐overexpression interfered with the interaction between NCOR2 and HSF4 (Figure 2I). As expected, BQ‐overexpression could reduce the amount of NCOR2 associated with HSE1 (Figure 2J) and favored the binding of HSF4 to HSE1 (Figure 2K). These results suggest a novel mechanism regarding the role of BQ on the transcriptional regulation of HIF‐1α as illustrated in Figure 2L.

The expression of KPNA1 and BQ in primary breast cancer samples was examined through immunohistochemistry (Figure 3A; Table S1). A positive correlation was observed between nuclear KPNA1 and nuclear BQ expression (Figure 3B). Patients with high KPNA1 had a higher nuclear BQ score (*p* < .05; Figure 3C). High nuclear KPNA1 expression was associated with poorer overall (*p* = .002; Figure 3D) and disease‐specific survival (*p* = .029; Figure 3E). Combined analysis for both KPNA1 and BQ nuclear expression showed even greater discrimination for poor overall survival (*p* = .0003; Figure 3F) and disease‐free survival (*p* = .007; Figure 3G). We also found that high nuclear expression of KPNA1 was associated with tamoxifen resistance (Figure 3H) and metastasis (Figure 3I). Cox‐regression analysis (Table 1) showed cases with high nuclear‐KPNA1 and high nuclear‐BQ was statistically significantly associated with poorer overall survival (RR = 3.832, 95% CI 1.758, 8.353; *p* = .001) and disease‐free survival (RR = 3.402, 95% CI 1.332, 8.693; *p* = .011).

**FIGURE 3:**
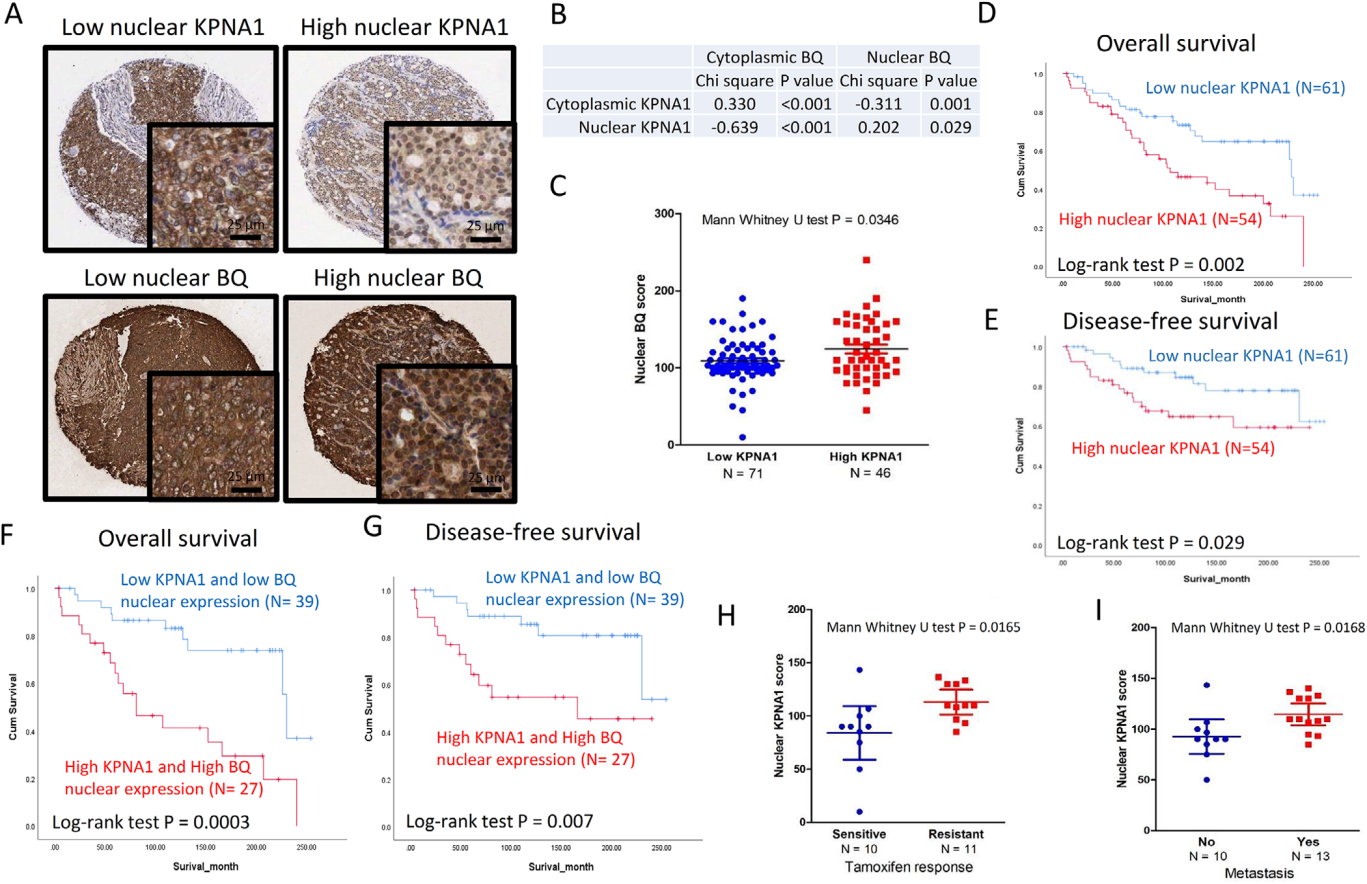
The clinical significance of KPNA1 and BQ in breast cancer. (A) Representative images of immunohistochemistry staining showing expression levels of KPNA1 and BQ in primary breast cancer samples in TMA. (B) Correlation between KPNA and BQ expression in the cytoplasm and in the nucleus assessed by Chi‐square test. (C) Mann–Whitney test showed that nuclear BQ score in low KPNA1 and high KPNA1 was different significantly (*p* = .035). Kaplan–Meier estimate showing breast cancer patients with high nuclear KPNA1 expression were associated with poorer (D) overall survival (*p* = .002) and (E) disease‐free survival outcome (*p* = .029) compared with the patients with low nuclear KPNA1 expression. Kaplan–Meier estimate showing breast cancer patients both high KPNA1 and high BQ nuclear expression were associated with poorer (F) overall survival (*p* = .0003) and (G) disease‐free survival (*p* = .007) outcomes compared with patients with both low KPNA1 and low BQ nuclear expression. Log‐rank test was employed to determine statistical significance. Mann–Whitney test showing high nuclear KPNA1 expression was associated with (H) tamoxifen resistance (*p* = .0165) and (I) metastasis (*p* = .0168). Nuclear KPNA1 and BQ scores where dichotomized at the median value

**TABLE 1:** Univariate analysis

		Overall survival		Disease‐specific survival	
Clinical–pathological parameters	No. of cases	RR (95% CI)	*p* Value	RR (95% CI)	*p* Value
Age	129	1.539 (0.899, 2.632)	.116	0.779 (0.388, 1.561)	.481
T stage	49	7.721 (2.552, 23.356)	**<.001**	5.261 (1.575, 17.572)	**.007**
Lymph‐node involvement	117	1.121 (0.649, 1.935)	.682	1.439 (0.686, 3.02)	.336
Tumor grade	119	0.876 (0.499, 1.535)	.643	2.143 (0.954, 4.816)	.065
Histological type	128	0.895 (0.439, 1.826)	.761	1.559 (0.475, 5.121)	.464
Estrogen receptor status	93	0.838 (0.393, 1.786)	.648	0.585 (0.262, 1.305)	.190
HER2 status	69	0.941 (0.426, 2.078)	.880	1.066 (0.431, 2.633)	.890
Triple negative	81	1.78 (0.721, 4.396)	.211	2.559 (0.991, 6.606)	.052
Tumor size	88	1.337 (0.652, 2.744)	.428	2.116 (0.761, 5.885)	.151
KPNA1 nuclear score	113	2.347 (1.339, 4.112)	**.003**	2.269 (1.064, 4.837)	**.034**
KPNA1 & BQ nuclear score	64	3.832 (1.758, 8.353)	**.001**	3.402 (1.332, 8.693)	**.011**

The value of the *P*‐value is 0.000295. (P < 0.001).

In conclusion, our investigation shows that nuclear import of BQ mediated by KPNA1 plays a critical role in modulating tamoxifen resistance. Nuclear‐BQ in competing with NCOR2 leads to the formation of defective corepressor complex, giving rise to upregulation of HIF‐1α. Thus, disruption of BQ nuclear import may be relevant to the development of therapeutic interventions in breast cancer. A recent finding that ERα repressor Neurofibromin (NF1) modulates tamoxifen resistance,^10^ further lends support to the importance of nuclear receptor corepressor in tamoxifen resistance. The possibility of other nuclear receptor corepressors involved remains to be investigated. These studies might help identify alternative therapeutic approaches for reducing tamoxifen resistance.

## CONFLICT OF INTEREST

The authors have no conflict of interest to declare. Ui‐Soon Khoo holds the patent of anti‐BQ323636.1 antibody (US Patent no: US 10823735; China Patent no: ZL201680051133.9).

## Supporting information

Supporting Information

Supporting Information

Supporting Information
