# Supplementary material for: KPNA1 regulates nuclear import of NCOR2 splice variant BQ323636.1 to confer tamoxifen resistance in breast cancer
Source: Clin Transl Med. 2021 Oct 12;11(10):e554. doi: 10.1002/ctm2.554 (PMC8506633; doi:10.1002/ctm2.554)
Supplement: Supplementary file 1 — Supporting Information [file CTM2-11-e554-s003.docx]

**Supplementary information**

**Abbreviations**

ATCC: American Type Culture Collection

BQ: BQ323636.1

ERα: Estrogen receptor α

HIF-1α: Hypoxia-inducible factor 1-alpha

HRE: Hypoxia Response Element

HSE: Heat shock Sequence Element

HSF2: Heat shock factor 2

HSF4: Heat shock factor 4

KPNA1 karyopherin subunit alpha 1

NCOR2: Nuclear receptor co-repressor 2

NLS: Nuclear localization signal

TAM: Tamoxifen

TMA: Tissue MicroArray

**Supplementary materials and methods**

**Cell culture, shRNA and siRNA**

Human breast cell lines MCF-7 and ZR-75 (tamoxifen sensitive cell lines) were purchased from American Type Culture Collection (ATCC) and were reauthenticated by short tandem repeat profiling.^1^ LCC2 is a tamoxifen-resistant cell line derived from MCF-7, kindly provided by Dr Robert Clarke (Georgetown University Medical School, Washington, DC; and used in our previous study^1^). MCF-7 and LCC2 cells were cultured and maintained in–Dulbecco's Modified Eagle Medium (DMEM, Gibco) supplemented with 10% fetal bovine serum (Gibco) and 1% penicillin/streptomycin (Gibco). ZR-75 cells were grown in Improved Minimum Essential Medium (IMEM, GIbco) with addition of 10% FBS and 1% P/S. All the cell lines were cultured in tissue culture incubator with 5% CO_2_ at 37°C. Cell lines used were confirmed mycoplasma-free. Mycoplasma screening was conducted by Faculty Core Facility (Li Ka Shing Faculty of Medicine, The University of Hong Kong). Hypoxic environment was created by culturing cells in 1% O_2_/5% CO_2_/balance N_2_ in a modulator incubator chamber at 37°C. MCF-7, ZR-75 and LCC2 were transfected with shCtrl (non-targeting shRNA; TR30021; OriGene), shKPNA1.1 (TL311853A, OriGene), shKPNA1.2 (TL311853B; OriGene), shKPNA1.3 (TL311853C; OriGene). Lipofectamine 2000 (Invitrogen) was employed for the transfection of plasmids. KPNA5 (assay ID: 11128) and KPNA6 (assay ID: 133901) siRNAs were purchase from ThermoFisher. 20 pmol of the siRNA was transfected into LCC2 cells with Oligofectamine (Invitrogen) according to manufacturer’s instruction. Tamoxifen (4-OHT, Sigma). AKT inhibitor (ab142088, Abcam), recombinant human Insulin-like Growth Factor 1 (IGF-1; PHG0078; ThermoFisher) were used.

**Stable cell line establishment**

After 72 hours post transfection, 0.5 µg/mL of puromycin (Gibco) was employed for selection of transfected cells. Fresh DMEM or IMEM with 10% FBS, 1% P/S and 1 µg/mL of puromycin was replaced every 72 hours. The selection was performed for six weeks. The cell lines were maintained in DMEM or IMEM with 10% FBS, 1% P/S and 1 µg/mL of puromycin.

**Molecular Cloning**

Classical NLS (PKKKRKV)^2^ and NES (CLPPLERLTR)^3^ was employed. The following linkers (5’🡪3’) were used: NLS linker-F (CCC GAA AAA AAA GCG CAA GGT GG), NLS linker-R (GAT CCC ACC TTG CGC TTT TTT TTC GGG GTA C), NES linker-F (CTG CTT GCC TCC TCT GGA GCG GCT CAC ACG CG), NES linker-R (GAT CCG CGT GTG AGC CGC TCC AGA GGA GGC AAG CAG GTA C) were annealed and subcloned into *pcDNA3.1-BQ* to generate *pcDNA3.1-BQ NLS* and *pcDNA3.1-BQ NES* by *Kpn1* (New England Biolabs) and *EcoR1* (New England Biolabs). NLS_BQ_ linker-F (CCC CCA GCG GCG GAG GCC CTC CCT GCT GTC TG), NLS_BQ_ linker-R (GAT CCA GAC AGC AGG GAG GGC CTC CGC CGC TGG GGG GTA C), NLS_BQ_ mt linker-F (CCC CCA GCG GCG GAG GCC CGC ACT GCT GTC TG) and NLS_BQ_ mt linker-R (GAT CCA GAC AGC AGT GCG GGC CTC CGC CGG TGG GGG GTA C) were subcloned into *pEGFPC1* (Clontech Laboratories) by *Kpn1* (New England Biolabs) and *BamH1* (New England Biolabs).

**Clinical specimens and Immunohistochemistry**

The TMA sections used included 137 breast cancer patients with pathological and clinical follow up data of over 10 years. The patients diagnosed between the years 1993 to 2003 with clinical follow up data were retrieved from the records of the Department of Pathology, Queen Mary Hospital of Hong Kong, with approval obtained from the Institutional Review Board of The University of Hong Kong (UW 08-147). Tamoxifen was used in the adjuvant setting for premenopausal women with confirmed ERα positive status. Out of 137 cases, 71 cases were ERα positive, 25 cases were ERα negative, and 41 cases with missing information (Table S1). Among the 71 ERα positive cases, 57 patients received tamoxifen as adjuvant therapy, 6 cases were untreated; with missing information for the remaining 8 cases. Tumor tissue was obtained from the surgical resection samples for construction of TMA blocks. Histological sections of all cases were reviewed by the pathologist, representative paraffin tumor blocks chosen as donor block for each case with the selected areas marked for construction of TMA blocks. Each case was constructed in triplicate in the TMA and average score of the triplicate was taken as the score. The IHC was performed as previously described.^4^ BQ323636.1 antibody (Anti-BQ, clone D12) was synthesized by InVivo BioTech Services GmbH (Germany), for which we hold the patent, US Patent no: US 10,823,735; China Patent no: ZL201680051133.9; details of antibody generation and quality control can be found in supplementary materials and methods of Gong et al^4^. Anti-BQ is now commercially available at Veritech Ltd, Hong Kong. Anti-BQ (1:50; D12; Versitech Ltd) and KPNA1 antibody (1:75; 2A4-1B5; Novus Biologicals) were used. Aperio ScanScope ® system (Leica Biosystems) was used to visualize and assess for BQ323636.1 (BQ) and KPNA1 expression and scored by two independent individuals. The intensities and percentages of nuclear staining were assessed as previously detailed.^5^ To avoid subjectivity in evaluation, the intensities and percentages of cytoplasmic staining and nuclear staining were separately scored in a semi-quantitative matter by two independent individuals. Cytoplasmic expression of KPNA1 and BQ were scored as follows. The intensity was scored as 0 = none, 1 = weak, 2 = moderate and 3 = strong. The percentage of cells stained was scored as 1 = less than 25%, 2 = 25% to 50%, 3 = 50% to 75% and 4 = more than 75%. The score was calculated as the product of the percentage score and the intensity score. Unlike cytoplasmic staining where a similar intensity was usually observed in cells within the case, variation of nuclear staining was observed, necessitating the use of the H-scoring system for assessment of nuclear score.^4, 6^ H-score = (1 x% of cells stained at intensity category 1) + (2 x % of cells stained at intensity category 2) + (3 x % of cells stained at intensity category 3). The median value was used to dichotomize the cutoff between low and high expression.

**Xenograft model**

Female nude mice at the age of 5 to 6 weeks were used for this study. On the day of inoculation, 1x10^6^ LCC2-shCtrl cells or 1x10^6^ LCC2-shKPNA1 cells were mixed with Matrigel (BD Bioscience) at a ratio of 1:1 and the 100 µl of the cell mixture were injected into abdominal mammary fat pad of the mice. When the tumors were palpable, mice were randomized into treatment and control groups where treatment group received 0.5 mg of tamoxifen dissolved in peanut oil (Sigma) by subcutaneous injection for twice per week. The injection started on day 7. The tumor sizes were measured regularly using caliper and the tumor volume was calculated as longest diameter x (shortest diameter)^2^/2. At the endpoint the experiments (52 days), mice were euthanized and tumors were harvested. All the procedures have been reviewed and approved by HKU Committee on the Use of Live Animals in Teaching and Research (CULATR No.:3259-14).

**Total RNA extraction, reverse transcription and real-time quantitative PCR**

TRIzol reagent (Invitrogen) was used for total RNA extraction following manufacturer’s protocol. Up to 0.5 μg of total RNAs were reverse transcribed into cDNA by SuperScript III reverse transcriptase (Invitrogen) following manufacturer’s protocol. Real-time PCR reaction was carried out with the ABI 7900HT Fast Real- time PCR system. ∆∆CT method was used to determine the relative gene expression. Cancer Pathway Finder PCR Array (PAHS-033Z; Qiagen) was employed. The following primers (5’🡪3’) were used: HIF-1α-F (ACC ACA GCT GAC CAG TTA TGA), HIF-1α-R (TGA GCC ACC AGT GTC CAA AA), HK-F (TCC CAT GTC CAA GAT CAC CC), HK-R (CTT GCC CCA TTA TCC CAT GC), ENO1-F (AAG TTT CCC CAT CTC CCA GG), ENO1-R (CAT TCA GGG CAG GTC ATT GG), LDHA-F (AGA GGC CCG TTT GAA GAA GA), LDHA-R (ACA ACA TGC ACA ACC TCC AC), PFK-F (CCA AAG AAC AGT GGT GGC TC), PFK-R (GTC AGA CAC TCC AGG GCT G), KPNA5-F (GCT GTT TGG GCA CTT GGT AA), KPNA5-R (CCC ACA CGG CAT TTC TTG TT), KPNA6-F (ATT CCG GAA ACT GCT CTC CA), KPNA6-R (TCA GAA ACT CCA CGA ACC GA), ACTIN-F (ATC GTG CGT GAC ATT AAG GAG AAG) and ACTIN-R (AGG AAG GAA GGC TGG AAG AGT G).

**Cell viability**

MTT assay (ThermoFisher) was performed. Clonogenic assay was performed. 0.01% of crystal violate was employed to stain the cell colonies. Cells were seeded on 24 wells. The colonies were stained with 0.01% crystal violet and counted under microscope. Colony with more than 50 cells was regarded as a colony. All experiments were performed in triplicate. Tecan Infinite F200 was used to record the absorbance.

**Nucleocytoplasmic fractionation and western blot**

Nucleocytoplasmic fractionation was performed by using NE-PER Nuclear and Cytoplasmic Extraction Reagents (Thermo Scientific). Cells were lysed using cell lysis buffer prepared by mixing 1x Cell Signaling buffer (Cell Signaling Technology), cOmplete Mini, EDTA-free protease inhibitor cocktail tablets (Roche), phenylmethanesulfonyl fluoride and PhosSTOP EASYPack tablets (Roche). The protein concentration of the cell lysates was determined by DC protein assay (BioRad). SDS-polyacrylamide gels were made in accordance with the protocol for Western blot analysis. 20 µg of total proteins were loaded in each of the wells of the gel. The following antibodies were used: anti-HIS tag (1:4,000; #2366; Cell Signaling Technology), anti-KPNA1 (1:1,000; PA5-21032; Invitrogen), anti-KPNA2 (1:1,000; NB100-79807; Novus Biologicals), anti-KPNA3 (1:1,000; NB100-81650; Novus Biologicals), anti-KPNA4 (1:1,000; NBP1-31260; Novus Biologicals), anti-KPNA5 (1:1,000; H00003841-M01; Novus Biologicals), anti-KPNA6 (1:1,000; NB100-93456; Novus Biologicals), anti-KPNA7 (1:1,000; NB100-93456; Novus Biologicals), anit-importin b1 (1:2,000; 05-1530; Merck), anti-p-AKT (1:1,000; 9271; Cell Signaling Technology); anti-GFP (1:5,000; #2955; Cell Signaling Technology), anti-phos-serine (1:500; #9631; Cell Signaling Technology ), anti-HIF-1α (1:1,000; #14179; Cell Signaling Technology), anti-HSF1 (1:2,000; ab2923; abcam), anti-HSF2 (1:500; sc-13517; Santa Cruz Biotechnology), anti-HSF4 (1:500; sc-398645; Santa Cruz Biotechnology), anti-NCOR2 (1:1000; ab2781l; Abcam), anti-β-tubulin (1:10,000; #2146; Cell Signaling Technology), anti-β-actin (1:10,000; sc-47778; Santa Cruz Biotechnology) and anti-Lamin B1 (1:4,000; sc-374015; Santa Cruz Biotechnology). The following reagents were used to generate signal: anti-mouse HRP (1:5000; P0447; Dako), anti-rabbit HRP (1:5000; P0260; Dako), anti-goat (1:2,000; sc-2354; Santa Cruz Biotechnology) and protein A/G HRP (1:8,000; 32490; Pierce). Band intensities from three independent sets of experiments were quantified using ImageJ. All quantified results were shown in Figures S13-S15. Data were expressed as target protein relative to the loading control. For protein expression analysis, following method was employed: (target protein – background) ÷ (loading control – background).

**Co-immunoprecipitation (CoIP) and Chromatin immunoprecipitation (ChIP)**

Cells were lysed in 440 µL of lysis buffer (20 mM Tris-Cl pH7.4 100 mM NaCl, 5 mM MgCl_2_, 0.5% NP-40 and 10% glycerol). 40 µL of the cell lysate was used as input. 200 µL of the lysate was either incubated with control anti-mouse IgG (1:200) or specific antibody (1:200) at 4°C overnight with rotation. 50 µL of Protein A/G agarose (Invitrogen) was used and incubated with the immunoprecipitant at room temperature for 2 hours with rotation. Western blot was performed. The signal was generated using protein A/G HRP. ChIP assay was performed using Magnetic ChIP Kit (Pierce). The following primers (5’🡪3’) were used: HSE1-F (ACT CTT TGC CAC GGA GCA CA), HSE1-R (GCT TGC AAA GTT GCC AAA GG), HSE2-F (TTG AGC CCA ACA AAG TAG CAT T), HSE2-R (CTT CTC TTC AGG CAT TTC CCA). Results from CoIP was expressed as the relative signal in elutant to input which was determined by the following method: (target antibody or anti-IgG – background) ÷ (input – background). For the proportion of protein 1 bound to protein 2, the results were determined by the following method: (elutant of protein 1 to the input of protein 1) ÷ (elutant of protein 2 to the input of protein 2).

**Luciferase reporter assay**

HIF-1α transcriptional activity was determined by HRE-luciferase (*pGL-HRE*; Plasmid #26731; Addgene).^7^ *pCMV*-Green Renilla Luc vector (*pCMV-Ren*; Thermo Scientific) was used for normalization. The ratio of *pGL-HRE* to *pCMV-Ren* was 100:1. Dual-Luciferase® Reporter Assay System (Promega) was used and the signal was captured and recorded by microplate reader Tecan Infinite F200.

**Statistical analysis**

All numerical data were processed in Excel (Microsoft), Prism5 (GraphPad) or SPSS25 (IBM). Data was expressed as mean ± SD from at least three independent experiments. The results of pathway enrichment analysis and differential gene expression profile were obtained from our previous study.^5^ Mann-Whitney U test or Students’ t test were performed to compare the variables of the 2 sample groups. One-way ANOVA was employed to determine the statistical significance for more 3 groups. The statistical significance between two groups was determined by Bonferroni's multiple comparison test. All tests were two-sided unless otherwise specified. Chi-square (χ^2^) test was used for hypothesis testing. Correlation with survival study of Tissue Microarray data was analyzed by Kaplan-Meier estimates followed by Log-rank test carried out by SPSS. Cox proportional hazards regression was used to estimate the association between clinical-pathological parameters, or BQ and KPNA1 scores with survival. Relative risk (RR) and 95% confidence interval (CI) were reported. The proportional-hazards assumption was tested using Omnibus test, and no major model violation was observed. P < 0.05 was considered statistically significant.

**Supplementary figure legends**

Figure S1 protein sequence alignment of NCOR2 and BQ. NCOR2 contains 2458 amino acids and BQ contains 362 amino acids. The alignment results showed that BQ can completely align to NCOR2 except the last 11 amino acids.

Figure S2 (A) Ectopic expression of BQ, BQ-NLS and BQ-NES in MCF-7 and ZR-75 cells. MCF-7 cells were transfected with the corresponding mammalian expression plasmids *pcDNA3.1-His-BQ*, *pcDNA3.1-His-BQ-NLS* and *pcDNA3.1-His-BQ-NES*. Classical NLS (PKKKRKV)^2^ and NES (CLPPLERLTR)^3^ were employed. Cells were harvested 48 hours post-transfection. Western blot was performed to determine the expression of the corresponding BQ proteins. Anti-His tag antibody was used. Actin was used as the loading control. Representative images were shown. (B) The subcellular localization of BQ, BQ-NLS and BQ-NES in MCF-7 and ZR-75. Confocal microscopy was performed 48 hours post-transfection on MCF-7 cells. Scale bar represents 5 μm. (C) Subcellular localization of BQ could affect cell viability. MTT assay was performed. Results were shown as mean ± SD from three independent experiments. Students’ t test was employed to determine the statistical significance between groups (BQ-NLS vs BQ and BQ-NES vs BQ). (D) Subcellular localization of BQ could affect the response to tamoxifen in MCF-7 and ZR-75 cells. Stable transfected cell lines were employed. The cells were treated with 5 μM, 7.5 μM, 10 μM and 15 μM of tamoxifen for 72 hours. EtOH was used as the solvent control. MTT assay was used to determine the cell viability. Results were shown as mean ± SD from three independent experiments. Students’ t test was employed to compare the statistical significance with EtOH group. All experiments were repeated at least three times. * and *** represent P < 0.05 and P < 0.001 respectively.

Figure S3 (A) Protein sequence of BQ. Putative nuclear localization signal is highlighted. The potential phosphorylation site onto the NLS is labelled in red. (B) Protein structures of NCOR2 (amino acid 33-366) and BQ (amino acid 3-357). The 3D structures were predicted using Phyre2.^8^ The templates for NCOR2 and BQ was c5lm2B and c1jchC respectively, which were obtained from Protein Data Bank. The amino acid surface accessibility was determined by RaptorX.^9^ B represents buried and E represents exposed. (C) KPNA1 could interact with NLS identified in BQ (NLS_BQ_). GFP was fused with NLS_BQ_. GFP and GFP- NLS_BQ_ were overexpressed in MCF-7 cells by transient transfection. Immunoprecipitation with anti-GFP was performed after 72 hours post-transfection.

Figure S4 (A) To determine the knockdown efficiency of two KPNA1 shRNAs (shKPNA1.1 and shKPNA1.2) in MCF-7-BQ and ZR-75-BQ. Western blot was performed to determine KPNA1 protein expression level. Tubulin was used as the loading control. Representative images were shown. (B) To confirm the knockdown of KPNA1 before nucleocytoplasmic fractionation and IF experiments were performed, knockdown of KPNA1 in MCF-7-BQ and ZR-75-BQ mediated by shKPNA1.1 was checked by Western blot on the whole cell lysates t. Actin was used as the loading control. (C) Knockdown of KPNA1 could diminish the nuclear import of BQ in both MCF-7-BQ and ZR-75-BQ cells. Confocal microscopy was performed 48 hours post-transfection on MCF-7-BQ and ZR-75-BQ cells. Scale bar represents 5 μm.

Figure S5 (A) Protein expression level of BQ in tamoxifen sensitive (MCF-7 and ZR-75) and tamoxifen resistant (LCC2) breast cancer cell lines. Western blot with anti-BQ antibody was used to detect BQ expression. Actin was used as loading control. (B) To determine the knockdown efficiency of two KPNA1 shKPNA (shKPNA1.a and shKPNA1.2) in LCC2 cells. Western blot was performed to determine KPNA1 protein expression level. Tubulin was used as the loading control. Representative images were shown. (C) To confirm the knockdown of KPNA1 before nucleocytoplasmic fractionation and IF experiments were performed, knockdown of KPNA1 in LCC2 mediated by shKPNA1.1 was checked by Western blot on the whole cell lysates.. Actin was used as the loading control. (D) Knockdown of KPNA1 could diminish the nuclear import of BQ in LCC2 cells. Confocal microscopy was performed 48 hours post-transfection on MCF-7-BQ and ZR-75-BQ cells. Scale bar represents 5 μm.

Figure S6 (A) Potential phosphorylation site on NLS_BQ_ was predicted using GPS web service. (B) Constructs were created expressing GFP fused with wild type NLS_BQ_ (PQRRRPSLLS; wtNLS_BQ_) and with mutant NLS_BQ_ (PQRRRPALLS; mtNLS_BQ_). The serine next to proline was converted to alanine in the mutant by site-directed mutagenesis. Expression of GFP with wild type NLS (GFP-wtNLS_BQ_) and mutant NLS (GFP-mtNLS_BQ_) in MCF-7 cells. The cells were transfected with the expression plasmids. The cells were harvested 48 hours post-transfection. Western blot was performed to determine the expression of GFP-wtNLS_BQ_ and GFP-mtNLS_BQ_. Tubulin was used as the loading control. Representative images were shown. (C) Comparing the interaction between KPNA1 and GFP-wtNLS_BQ_ versus GFP-mtNLS_BQ_. MCF-7 and ZR-75 cells were transiently transfected by pEGFP-wtNLS_BQ_ and pEGFP-mtNLS_BQ_. Immunoprecipitation with anti-GFP was performed after 72 hours post-transfection. (D) Inhibition of AKT could compromise the interaction between KPNA1 and GFP-wtNLS_BQ_. 1 μM of the AKT inhibitor was used for treating the cells for 72 hours. Immunoprecipitation with anti-GFP was performed. (E) The effect of IGF-1 treatment on AKT activation in LCC2. The cells were treated with the indicated amount of IGF-1 for 24 hours. Western blot was employed to determine the level of phos-AKT. Actin was used as loading control.

Figure S7 (A) Overexpression of BQ in MCF-7 and ZR-75 cell lines. MCF-7 and ZR-75 cells were stably transfected with control pcDNA3.1 plasmid and BQ overexpression plasmid pcDNA3.1-His-BQ. Western blot with anti-BQ antibody was used to detect BQ overexpression. Actin was used as loading control. (B) Knockdown efficiency of KPNA1 mediated by shRNA. Three independent plasmids which expressed specific KPNA1 targeting shRNA (shKPNA1.1, shKPNA1.2, shKPNA1.3) and one plasmid which expressed non-targeting shRNA (shCtrl) were employed to establish the stable cell lines. qPCR was employed to determine the knockdown efficiency of the shRNA. Actin was used as the internal control. Results were shown as mean ± SD from three independent experiments. Students’ t test was employed to determine the statistical significance compared with the control. *** represents P < 0.001. Down-regulation of KPNA1 could confer tamoxifen sensitivity in (C) MCF-7-BQ, (D) ZR-75-BQ and (E) LCC2 as revealed by MTT assay. Two independent stable KPNA1 knockdown cell lines were generated by using two independent KPNA1 shRNA expressing plasmids. Parental MCF-7-BQ/ZR-75-BQ/LCC2 and cells which expressed non-targeting shRNA (shCtrl) were used as controls. The cells were treated with 5 μM of tamoxifen (TAM) or ethanol (EtOH) for 72 hours. MTT assay was performed to determine cell viability. Results were shown as mean ± SD from three independent experiments. Students’ t test was employed to determine the statistical significance when compared with untreated control (-). Down-regulation of KPNA1 could confer tamoxifen sensitivity in (F) MCF-7-BQ, (G) ZR-75-BQ and (H) LCC2 as revealed by clonogenic assay. The cells were treated with 5 μM of tamoxifen (TAM) or ethanol (EtOH) for 14 days. 0.01% crystal violet was used to stain the cells. Representative images were shown. Results were shown as mean ± SD from three independent experiments. * and *** represent P < 0.05 and P < 0.001 respectively.

Figure S8 (A) Comparing the identity of protein sequence with KPNA1 (NP_002255.3). Protein sequences of KPNA1 (NP_002255.3), KPNA2 (NP_001307540.1), KPNA3 (NP_002258.2), KPNA4 (NP_002259.1), KPNA5 (NP_001353233.1), KPNA6 (NP_036448.1) and KPNA7 (NP_001139187.1) were obtained from NCBI. Sequence alignment was employed to determine the protein identity. Knockdown efficiency of (B) siKPNA5 and (C) KPNA6 in LCC2 cells. LCC2 cells were treated with 20 pmol of siCtrl, siKPNA5 or siKPNA6 for 48 hours. qPCR was employed to determine the expression of KPNA5 and KPNA6. Actin was used as the internal control. Results were shown as mean ± SD from three independent experiments. Students’ t test was employed to determine the statistical significance compared with siCtrl. *** represents P < 0.001. (D) Knockdown of KPNA5 and KPNA6 did not affect tamoxifen resistance in LCC2. LCC2 cells were treated with 20 pmol of the corresponding siRNA for 48 hours and then treated with 5 μM of tamoxifen (TAM). MTT assay was performed 72 hours of TAM treatment. Results were shown as mean ± SD from three independent experiments.

Figure S9 Knockdown efficiency of siRNA targeting KPNA1 in (A) MCF-7 and (B) ZR-75. The cells were transfected with the indicated amount of KPNA1 targeting siRNA. qPCR was performed after 72 hours post-transfection. Actin was used as the internal control. Results were shown as mean ± SD from three independent experiments. Students’ t test was employed to determine the statistical significance compared with the control. *, **, *** represent P < 0.05, P < 0.01 and P < 0.001 respectively. (C) Protein expression of KPNA1 was reduced significantly. MCF-7 and ZR-75 cells were transfected with 20 pmol of either non-targeting siRNA or siKNPA1. Western blot was performed after 72 hours post-transfection. Actin was used as the loading control. Representative images were shown.

Figure S10 (A) Knockdown of KPNA1 could reduce the protein level of HIF-1α in LCC2. Western blot was employed to detect the expression of HIF-1α. Actin was used as loading control. (B) Knockdown of KPNA1 could reduce HIF-1α activity in LCC2. Luciferase reporter assay with HIF-1α response element (HRE) was performed. The cells were cultured in non-hypoxic conditions. Results were shown as mean ± SD from three independent experiments. Students’ t test was employed to determine the statistical significance compared with the control. *** represents P < 0.001.

Figure S11 The structure of HIF-1α promoter. HSE is the DNA region for interacting with HSF. The diagram is adapted from published study.^10^

Figure S12 Quantification of protein band intensity shown in Figure 1.

(A) Quantification of the expression of the candidate proteins in the elutants relative to input of MCF-7 in Figure 1A.

(B) Quantification of the expression of the candidate proteins in the elutants relative to input of ZR-75 in Figure 1A.

(C) Quantification of the expression of BQ-His relative to LaminB1 in the nuclear fraction of MCF-7-BQ in Figure 1B.

(D) Quantification of the expression of BQ-His relative to tubulin in the cytoplasmic fraction of MCF-7-BQ in Figure 1B.

(E) Quantification of the expression of BQ-His relative to LaminB1 in the nuclear fraction of ZR-75-BQ in Figure 1C.

(F) Quantification of the expression of BQ-His relative to tubulin in the cytoplasmic fraction of ZR-75-BQ in Figure 1C.

(G) Quantification of the expression of endogenous BQ relative to LaminB1 in the nuclear fraction of LCC2 in Figure 1D.

(H) Quantification of the expression of endogenous BQ relative to tubulin in the cytoplasmic fraction of LCC2 in Figure 1D.

(I) Quantification of the relative proportion of KPNA1 bound to BQ in MCF-7-BQ treated with AKT inhibitor in Figure 1E.

(J) Quantification of the relative proportion of KPNA1 bound to BQ in ZR-75-BQ treated with AKT inhibitor in Figure 1E.

(K) Quantification of the expression of BQ-His relative to LaminB1 in nuclear fraction of MCF-7-BQ in Figure 1F.

(L) Quantification of the expression of BQ-His relative to tubulin in cytoplasmic fraction of MCF-7-BQ in Figure 1F.

(M) Quantification of the expression of BQ-His relative to LaminB1 in nuclear fraction of ZR-75-BQ in Figure 1F.

(N) Quantification of the expression of BQ-His relative to tubulin in cytoplasmic fraction of ZR-75-BQ in Figure 1F.

(O) Quantification of the expression of endogenous BQ relative to LaminB1 in nuclear fraction of LCC2 treated with10 nM of IGF-1 in Figure 1G.

(P) Quantification of the expression of endogenous BQ relative to tubulin in cytoplasmic fraction of LCC2 treated with 10 nM of IGF-1 in Figure 1G.

Students’ t test was employed for statistical analysis for two groups. One-way ANOVA followed by Bonferroni's multiple comparison test was employed to determine statistical significance for a pair of groups in three more data sets. *, **, *** represent P < 0.05, P < 0.01 and P < 0.001, respectively.

Figure S13 Quantification of protein band intensity shown in Figure 2.

(A) Quantification of the expression of HIF-1α relative to actin in MCF-7 with or without BQ overexpression under normal and hypoxic condition in Figure 2B.

(B) Quantification of the expression of HIF-1α relative to actin in ZR-75 with or without BQ overexpression under normal and hypoxic condition Figure 2B.

(C) Quantification of the expression of HIF-1α relative to tubulin in MCF-7 and MCF-7-BQ treated with siRNA against KPNA1 in Figure 2E.

(D) Quantification of the expression of HIF-1α relative to tubulin in ZR-75 and ZR-75-BQ treated with siRNA against KPNA1 in Figure 2E.

(E) Quantification of the amount of HSF2 and HSF4 in the elutants relative to input in MCF-7 in Figure 2G.

(F) Quantification of the amount of HSF2 and HSF4 in the elutants relative to input in ZR-75 in Figure 2G.

(G) Quantification of the proportion of BQ bound to NCOR2 in MCF-7 and MCF-7-BQ in Figure 2I.

(H) Quantification of the proportion of HSF4 bound to NCOR2 in MCF-7 and MCF-7-BQ in Figure 2I.

(I) Quantification of the proportion of BQ bound to NCOR2 in ZR-75 and ZR-75-BQ in Figure 2I.

(J) Quantification of the proportion of HSF4 bound to NCOR2 in ZR-75 and ZR-75-BQ in Figure 2I.

Students’ t test was employed for statistical analysis for two groups. One-way ANOVA followed by Bonferroni's multiple comparison test was employed to determine statistical significance for a pair of groups in three data sets. *** represents P < 0.001.

Figure S14 Quantification of protein band intensity shown in supplementary figures.

(A) Quantification of the expression of KPNA1 relative to tubulin in MCF-7-BQ in Figure S4A.

(B) Quantification of the expression of KPNA1 relative to tubulin in ZR-75-BQ in Figure S4A.

(C) Quantification of the expression of KPNA1 relative to tubulin in the whole cell lysates of MCF-7-BQ prior to nucleocytoplasmic fractionation and IF in Figure S4B.

(D) Quantification of the expression of KPNA1 relative to tubulin in in the whole cell lysates of ZR-75-BQ prior to nucleocytoplasmic fractionation and IF in Figure S4B.

(E) Quantification of the expression of endogenous BQ relative to actin in MCF-10A, MCF-7, ZR-75 and LCC2 in Figure S5A.

(F) Quantification of the expression of KPNA1 in LCC2, LCC2-shCtrl, LCC2-shKPNA1.1 and LCC2-shKPNA1.2 in Figure S5B.

(G) Quantification of the expression of KPNA1 relative to actin in the whole cell lysates of LCC2 prior to nucleocytoplasmic fractionation and IF in Figure S5C.

(H) Quantification of the proportion of GFP-wtNLS_BQ_ and GFP-mtNLS_BQ_ bound to KPNA1 in MCF-7 in Figure S6C.

(I) Quantification of the proportion of GFP-wtNLS_BQ_ and GFP-mtNLS_BQ_ bound to KPNA1 in ZR-75 in Figure S6C.

(J) Quantification of the proportion of GFP-wtNLS_BQ_ bound to KPNA1 in MCF-7 treated with AKT inhibitor in Figure S6D.

(K) Quantification of the proportion of GFP-wtNLS_BQ_ bound to KPNA1 in ZR-75 treated with AKT inhibitor in Figure S6D.

(L) Quantification of the expression of phosphorylated AKT (p-AKT) relative to actin in LCC2 treated with IGF-1 in Figure S6E.

(M) Quantification of the expression of BQ relative to actin in MCF-7 in Figure S7A.

(N) Quantification of the expression of BQ relative to actin in ZR-75 in Figure S7A.

(O) Quantification of the expression of KPNA1 relative to actin in MCF-7 treated with siRNA against KPNA1 in Figure S9C.

(P) Quantification of the expression of KPNA1 relative to actin in ZR-75 treated with siRNA against KPNA1 in Figure S9C.

(Q) Quantification of the expression of HIF-1α relative to actin in LCC2, LCC2-shCtrl, LCC2-shKPNA1.1 and LCC2-shKPNA1.2 in Figure S10A.

Students’ t test was employed for statistical analysis for two groups. One-way ANOVA followed by Bonferroni's multiple comparison test was employed to determine statistical significance for a pair of groups in three or more data sets. *, **, *** represent P < 0.05, P < 0.01 and P < 0.001, respectively.

**References**

1. Zhang, L.*, et al*. SpliceArray Profiling of Breast Cancer Reveals a Novel Variant of NCOR2/SMRT That Is Associated with Tamoxifen Resistance and Control of ERα Transcriptional Activity. *Cancer Research*. **73**, 246-255 (2013).

2. Kalderon, D., Roberts, B.L., Richardson, W.D. & Smith, A.E. A short amino acid sequence able to specify nuclear location. *Cell*. **39**, 499-509 (1984).

3. Fornerod, M., Ohno, M., Yoshida, M. & Mattaj, I.W. CRM1 is an export receptor for leucine-rich nuclear export signals. *Cell*. **90**, 1051-1060 (1997).

4. Gong, C.*, et al*. BQ323636.1, a Novel Splice Variant to NCOR2, as a Predictor for Tamoxifen-Resistant Breast Cancer. *Clin Cancer Res*. **24**, 3681-3691 (2018).

5. Tsoi, H., Man, E.P.S., Chau, K.M. & Khoo, U.S. Targeting the IL-6/STAT3 Signalling Cascade to Reverse Tamoxifen Resistance in Estrogen Receptor Positive Breast Cancer. *Cancers (Basel)*. **13**, (2021).

6. Detre, S., Saclani Jotti, G. & Dowsett, M. A "quickscore" method for immunohistochemical semiquantitation: validation for oestrogen receptor in breast carcinomas. *Journal of clinical pathology*. **48**, 876-878 (1995).

7. Emerling, B.M., Weinberg, F., Liu, J.L., Mak, T.W. & Chandel, N.S. PTEN regulates p300-dependent hypoxia-inducible factor 1 transcriptional activity through Forkhead transcription factor 3a (FOXO3a). *P Natl Acad Sci USA*. **105**, 2622-2627 (2008).

8. Kelley, L.A., Mezulis, S., Yates, C.M., Wass, M.N. & Sternberg, M.J.E. The Phyre2 web portal for protein modeling, prediction and analysis. *Nat Protoc*. **10**, 845-858 (2015).

9. Kallberg, M.*, et al*. Template-based protein structure modeling using the RaptorX web server. *Nat Protoc*. **7**, 1511-1522 (2012).

10. Chen, R., Liliental, J.E., Kowalski, P.E., Lu, Q. & Cohen, S.N. Regulation of transcription of hypoxia-inducible factor-1 alpha (HIF-1 alpha) by heat shock factors HSF2 and HSF4. *Oncogene*. **30**, 2570-2580 (2011).
