# Supplementary material for: KPNA1 regulates nuclear import of NCOR2 splice variant BQ323636.1 to confer tamoxifen resistance in breast cancer
Source: Clin Transl Med. 2021 Oct 12;11(10):e554. doi: 10.1002/ctm2.554 (PMC8506633; doi:10.1002/ctm2.554)

Figure S1

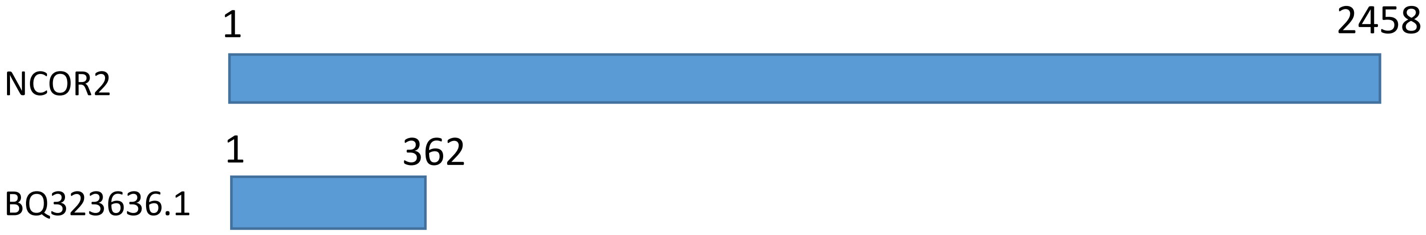


```
CLUSTAL O(1.2.4) multiple sequence alignment


NCOR2           MSGSTQPVAQTWRATEPRYPPHSLSYPVQIARTHTDVGLLEYQHHSRDYASHLSPGSIIQ 60
BQ323636.1      MSGSTQPVAQTWRATEPRYPPHSLSYPVQIARTHTDVGLLEYQHHSRDYASHLSPGSIIQ 60
                ************************************************************

NCOR2           PQRRRPSLLSEFQPGNERSQELHLRPESHSYLPELGKSEMEFIESKRPRLELLPDPLLRP 120
BQ323636.1      PQRRRPSLLSEFQPGNERSQELHLRPESHSYLPELGKSEMEFIESKRPRLELLPDPLLRP 120
                ************************************************************

NCOR2           SPLLATGQPAGSEDLTKDRSLTGKLEPVSPPSPPHTDPELELVPPRLSKEELIQNMDRVD 180
BQ323636.1      SPLLATGQPAGSEDLTKDRSLTGKLEPVSPPSPPHTDPELELVPPRLSKEELIQNMDRVD 180
                ************************************************************

NCOR2           REITMVEQQISKLKKKQQQLEEEAAKPPEPEKPVSPPPIESKHRSLVQIIYDENRKKAEA 240
BQ323636.1      REITMVEQQISKLKKKQQQLEEEAAKPPEPEKPVSPPPIESKHRSLVQIIYDENRKKAEA 240
                ************************************************************

NCOR2           AHRILEGLGPQVELPLYNQPSDTRQYHENIKINQAMRKKLILYFKRRNHARKQWEQKFCQ 300
BQ323636.1      AHRILEGLGPQVELPLYNQPSDTRQYHENIKINQAMRKKLILYFKRRNHARKQWEQKFCQ 300
                ************************************************************

NCOR2           RYDQLMEAWEKKVERIENNPRRRAKESKVREYYEKQFPEIRKQRELQERMQRVGQRGSGL 360
BQ323636.1      RYDQLMEAWEKKVERIENNPRRRAKESKVREYYEKQFPEIRKQRELQERMQRTWRSRCAS 360
                ****************************************************. :  .. 

NCOR2           SMSAARSEHEVSEIIDGLSEQENLEKQMRQLAVIPPMLYDADQQRIKFINMNGLMADPMK 420
BQ323636.1      WP---------------------------------------------------------- 362
```

Figure S2

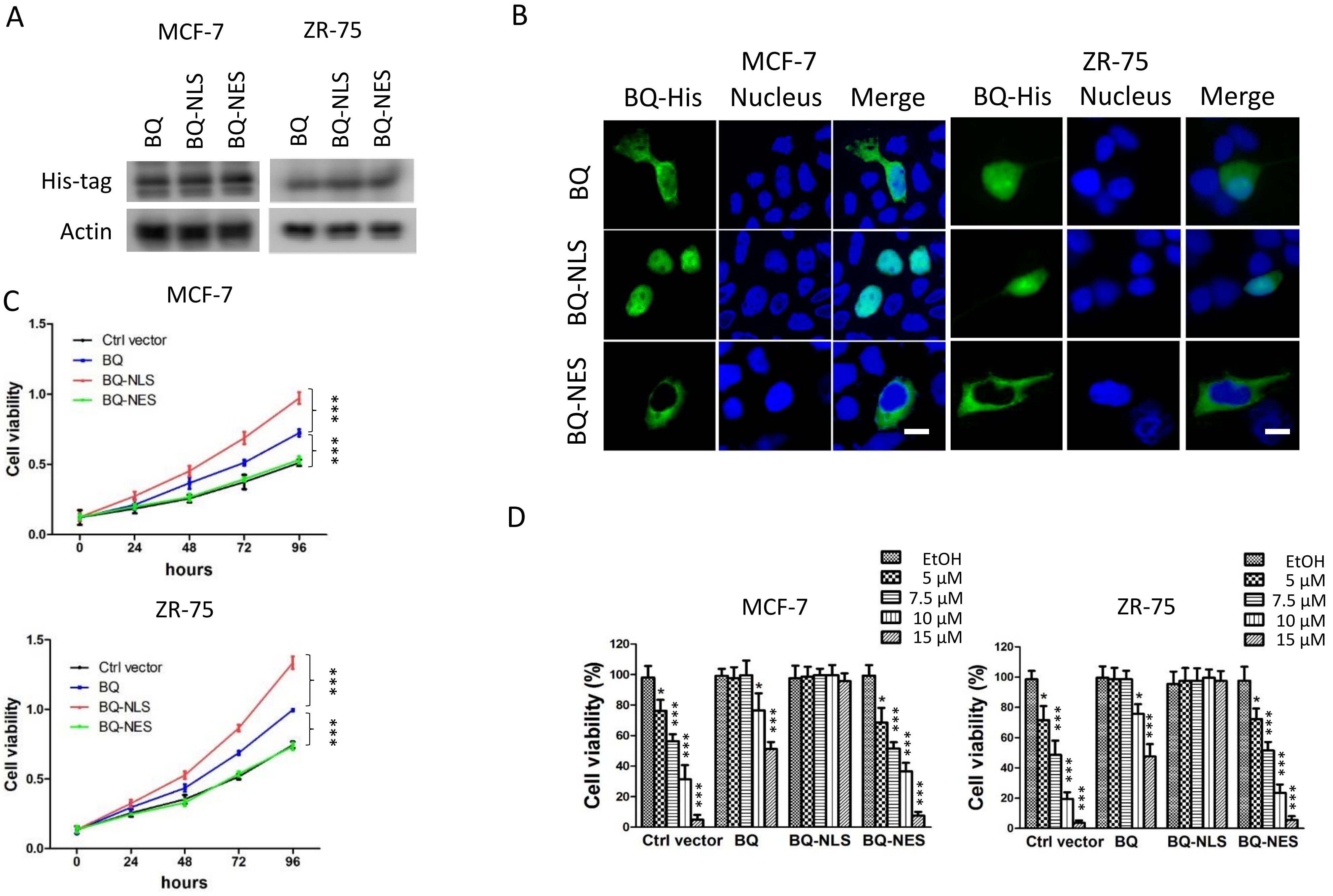
A
MCF-7
ZR-75
BQ
BQ-NLS
BQ-NES
His-tag
Actin
B
MCF-7
ZR-75
BQ-His
Nucleus
Merge
BQ
BQ-NLS
BQ-NES
C
MCF-7
Ctrl vector
BQ
BQ-NLS
BQ-NES
Cell viability
hours
ZR-75
***
D
MCF-7
ZR-75
EtOH
5 μM
7.5 μM
10 μM
15 μM
Cell viability (%)
Ctrl vector
BQ
BQ-NLS
BQ-NES

Figure S3

A **Protein sequence of BQ**

MSGSTQPVAQTWRATEPRYPPHSLSYPVQIARTHTDVGLLEYQHHSRDYASHLSPGSIIQ**PQRRRPSLLS**EFQPGNERSQELHLRPESHSYLPEL
GKSEMEFIESKRPRLELLPDPLLRPSPLLATGQPAGSEDLTKDRSLTGKLEPVSPPSPPHTDPELELVPPRLSKEELIQNMDRVDREITMVEQQISK
LKKKQQQLEEEAAKPPEPEKPVSPPPIESKHRSLVQIIYDENRKKAEAAHRILEGLGPQVELPLYNQPSDTRQYHENIKINQAMRKKLILYFKRRN
HARKQWEQKFCQRYDQLMEAWEKKVERIENNPRRRAKESKVREYYEKQFPEIRKQRELQERMQRTWRSRCASWP*

Key:
Bold and underline: NLS_{BQ}; potential nuclear localization signal (NLS) on BQ
Red: Potential phosphorylation site by AKT

B

NCOR2 (amino acid 33-366)
Confidence: 95.24%

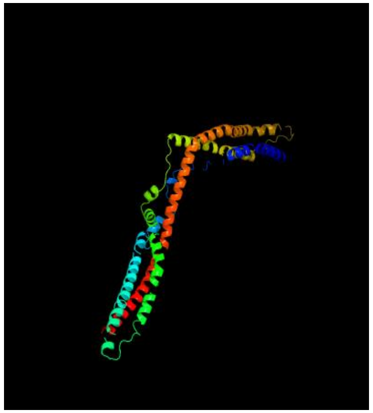

Position	Amino acid	Class assignment	Relative Surface Accessibility
61	P	E	0.425
62	Q	E	0.465
63	R	B	0.202
64	R	B	0.271
65	R	B	0.265
66	P	B	0.242
67	S	B	0.348
68	L	E	0.291
69	L	B	0.080
70	S	B	0.132

BQ (amino acid 3-357)
Confidence: 81.63%

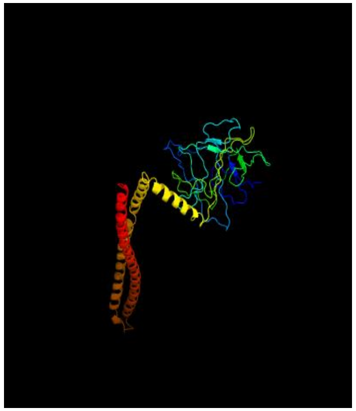

Position	Amino acid	Class assignment	Relative Surface Accessibility
61	P	E	0.425
62	Q	E	0.465
63	R	E	0.425
64	R	E	0.335
65	R	B	0.265
66	P	B	0.242
67	S	E	0.348
68	L	E	0.291
69	L	B	0.080
70	S	E	0.374

C

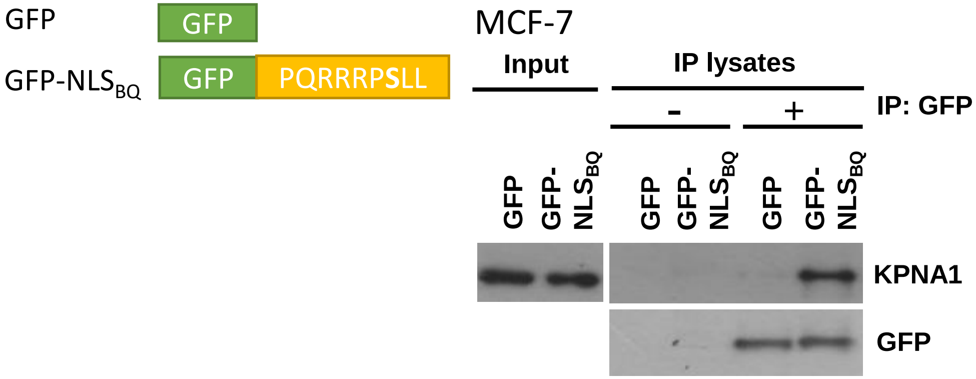

Figure S4

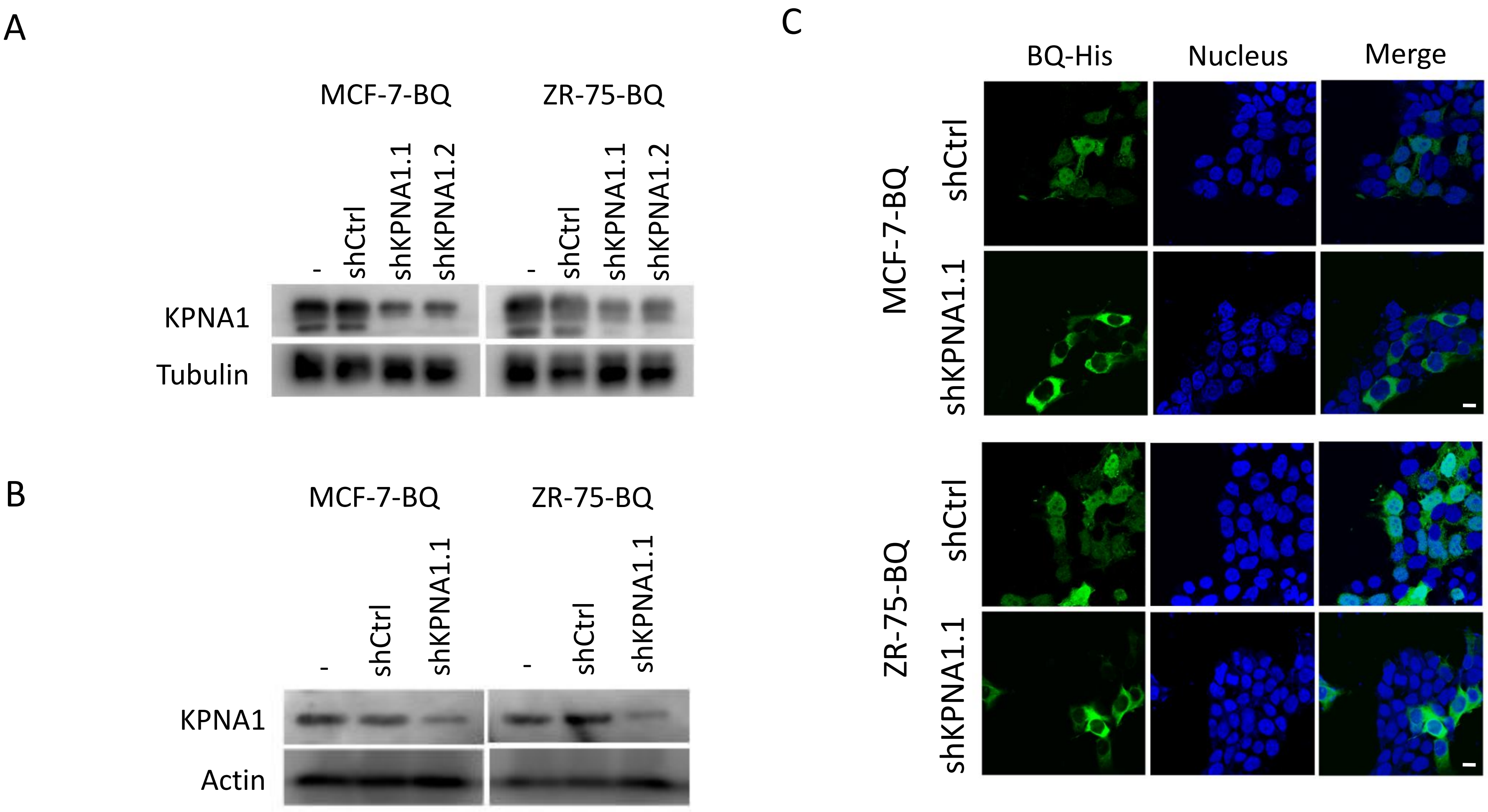
A
MCF-7-BQ
ZR-75-BQ
-
shCtrl
shKPNA1.1
shKPNA1.2
KPNA1
Tubulin
B
MCF-7-BQ
ZR-75-BQ
-
shCtrl
shKPNA1.1
KPNA1
Actin
C
BQ-His
Nucleus
Merge
MCF-7-BQ
shCtrl
shKPNA1.1
ZR-75-BQ
shCtrl
shKPNA1.1

Figure S5

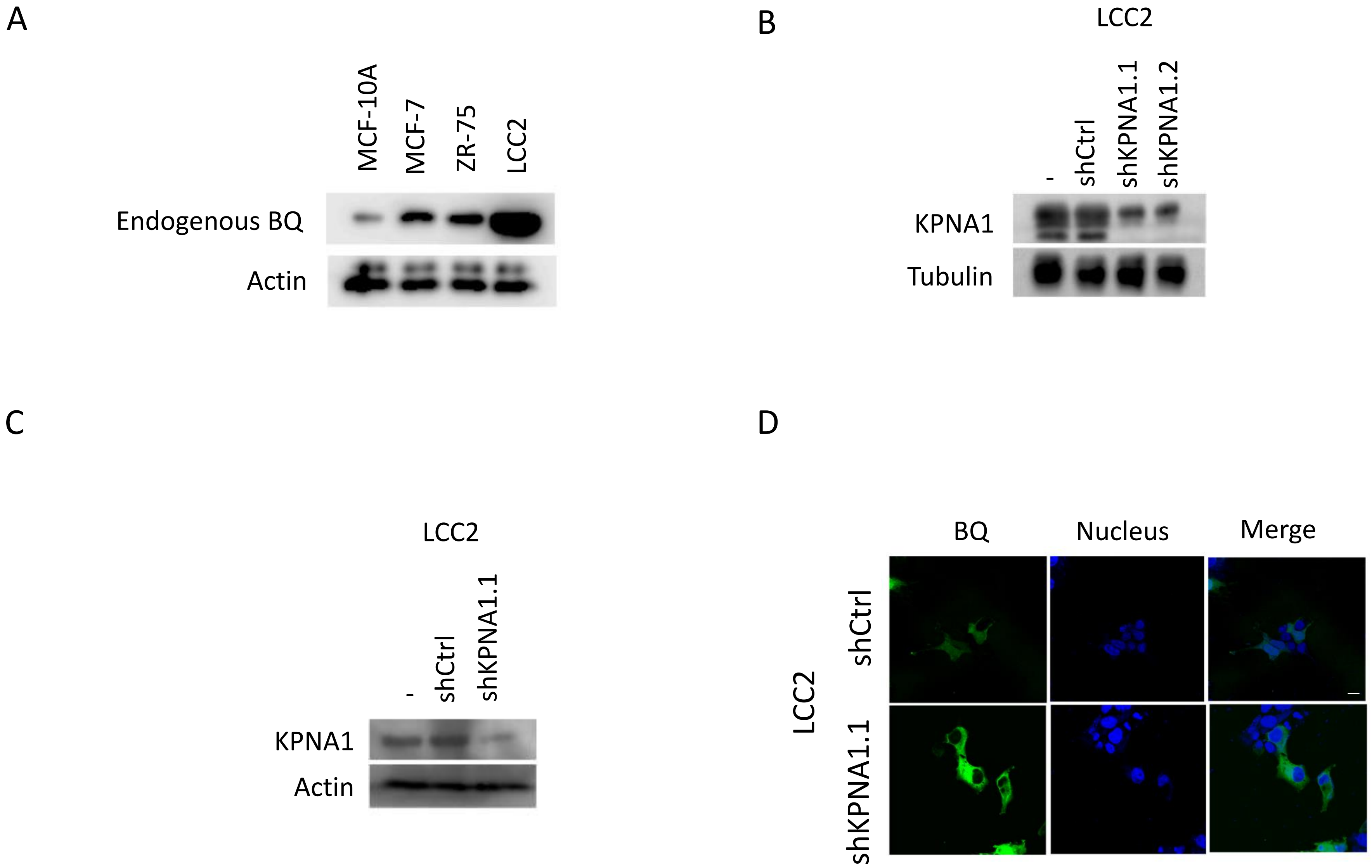
A
MCF-10A
MCF-7
ZR-75
LCC2
Endogenous BQ
Actin
B
LCC2
-
shCtrl
shKPNA1.1
shKPNA1.2
KPNA1
Tubulin
C
LCC2
-
shCtrl
shKPNA1.1
KPNA1
Actin
D
BQ
Nucleus
Merge
LCC2
shCtrl
shKPNA1.1

Figure S6

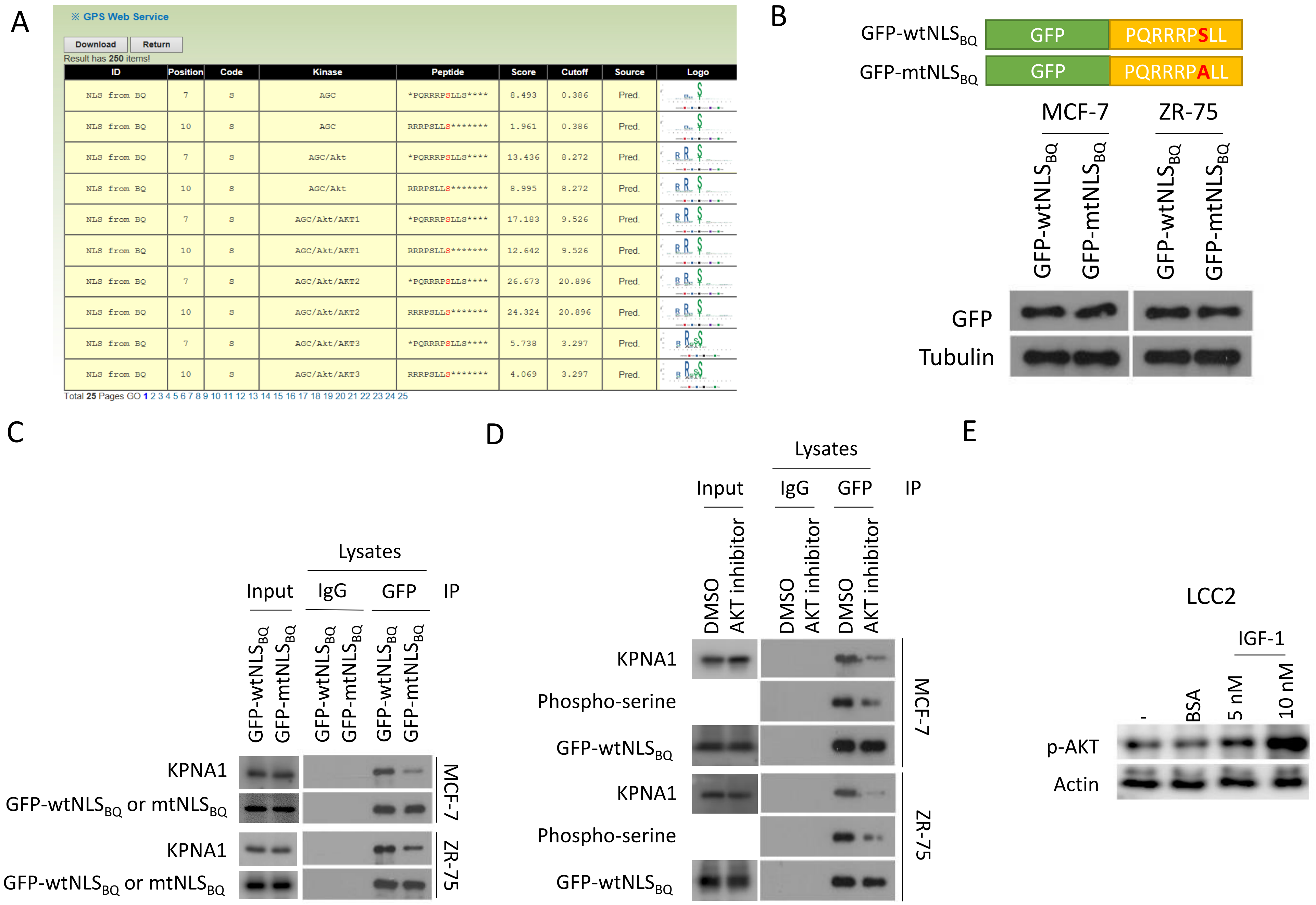

ID	Position	Code	Kinase	Peptide	Score	Cutoff	Source	Logo
NLS from BQ	7	S	AGC	*PQRRRPSLLS****	8.493	0.386	Pred.	
NLS from BQ	10	S	AGC	RRRPSLLS*******	1.961	0.386	Pred.	
NLS from BQ	7	S	AGC/Akt	*PQRRRPSLLS****	13.436	8.272	Pred.	
NLS from BQ	10	S	AGC/Akt	RRRPSLLS*******	8.995	8.272	Pred.	
NLS from BQ	7	S	AGC/Akt/AKT1	*PQRRRPSLLS****	17.183	9.526	Pred.	
NLS from BQ	10	S	AGC/Akt/AKT1	RRRPSLLS*******	12.642	9.526	Pred.	
NLS from BQ	7	S	AGC/Akt/AKT2	*PQRRRPSLLS****	26.673	20.896	Pred.	
NLS from BQ	10	S	AGC/Akt/AKT2	RRRPSLLS*******	24.324	20.896	Pred.	
NLS from BQ	7	S	AGC/Akt/AKT3	*PQRRRPSLLS****	5.738	3.297	Pred.	
NLS from BQ	10	S	AGC/Akt/AKT3	RRRPSLLS*******	4.069	3.297	Pred.	

Figure S7

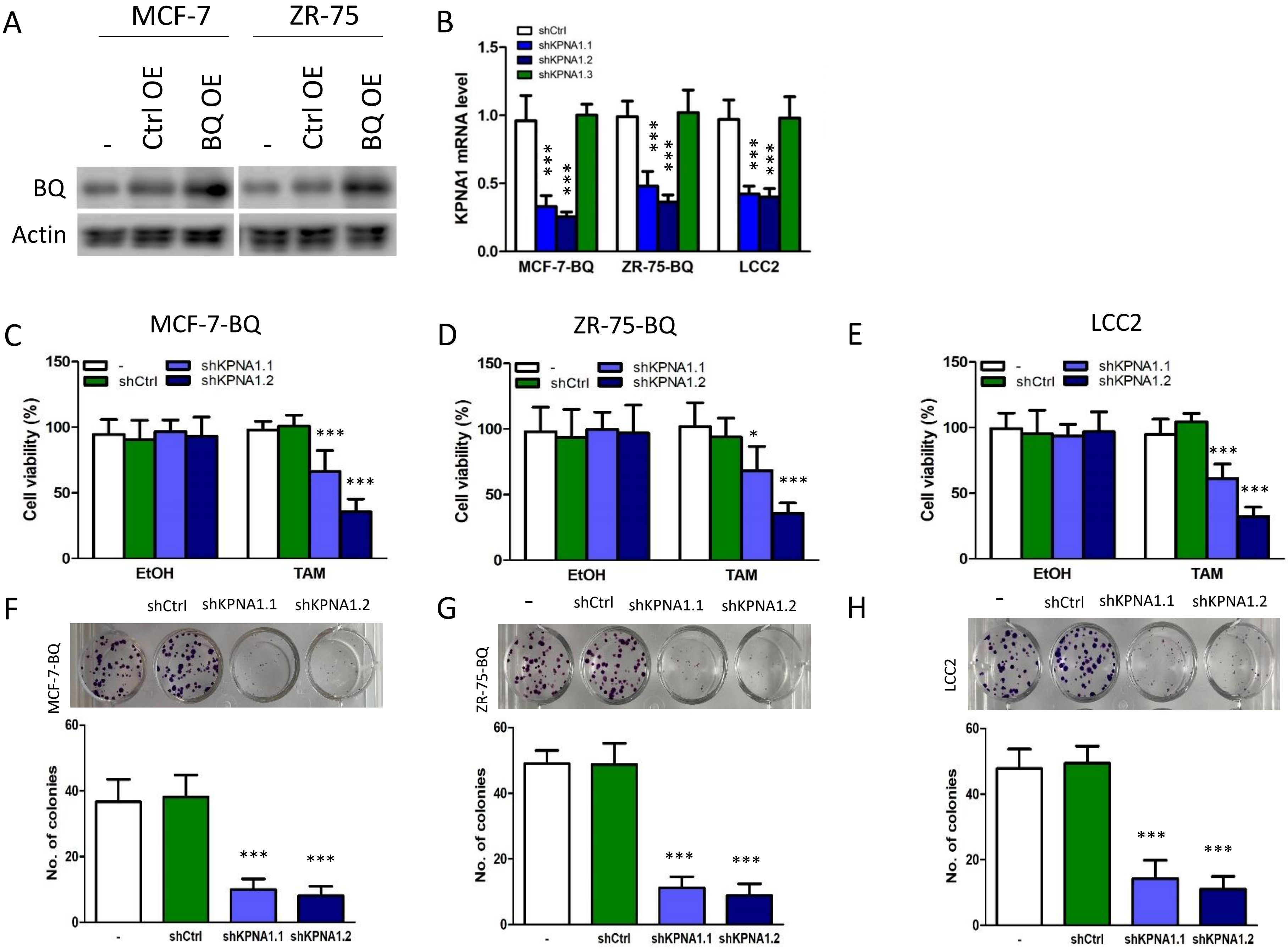
A
MCF-7
ZR-75
-
Ctrl OE
BQ OE
BQ
Actin
B
shCtrl
shKPNA1.1
shKPNA1.2
shKPNA1.3
KPNA1 mRNA level
MCF-7-BQ
ZR-75-BQ
LCC2
C
MCF-7-BQ
D
ZR-75-BQ
E
LCC2
shKPNA1.1
shKPNA1.2
Cell viability (%)
EtOH
TAM
F
G
H
No. of colonies

Figure S8

A

	Identity
KPNA2	45%
KPNA3	47%
KPNA4	47%
KPNA5	77%
KPNA6	81%
KPNA7	41%

B

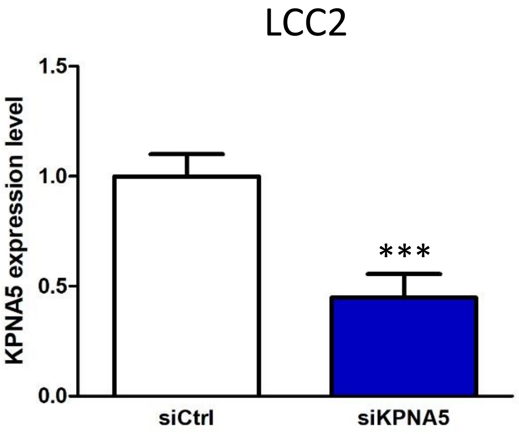

C

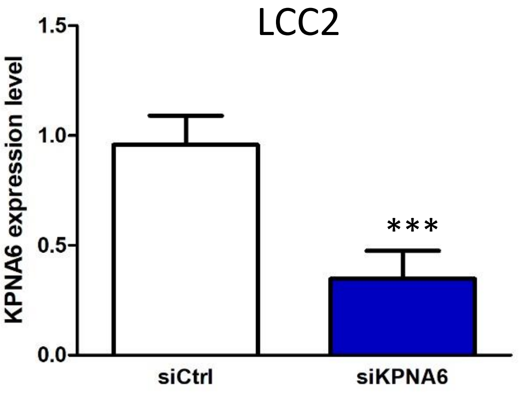

D

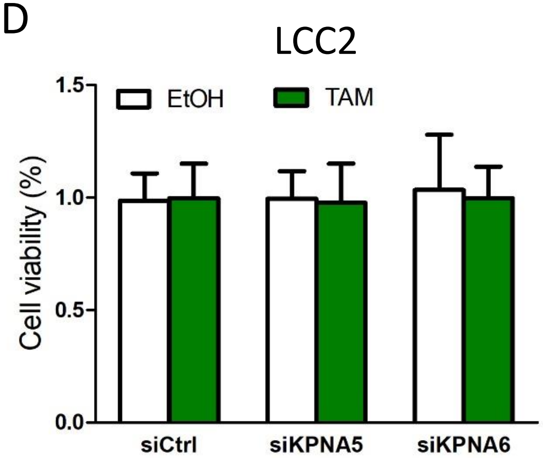

Figure S9

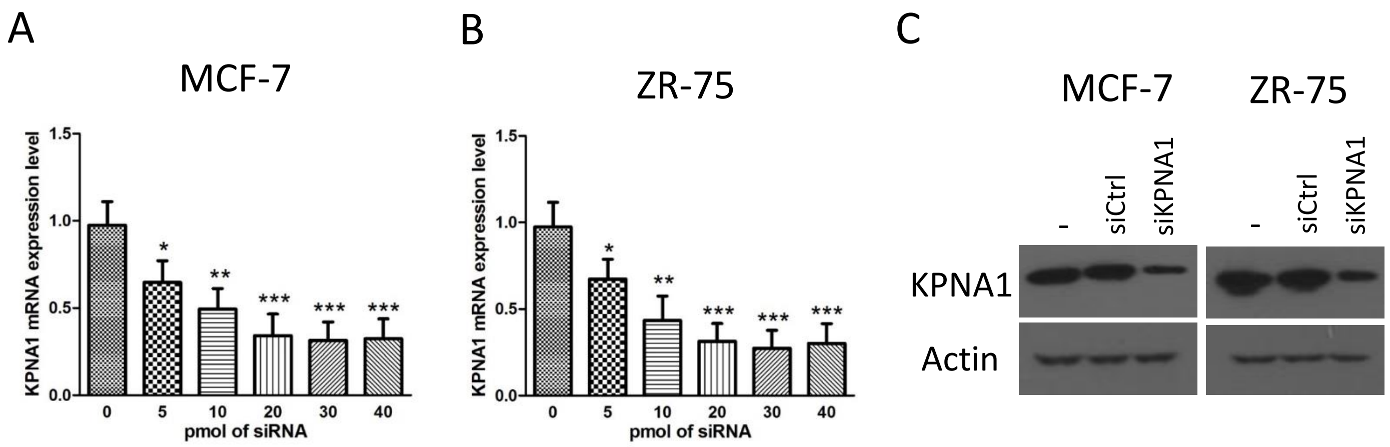
A
MCF-7
KPNA1 mRNA expression level
1.5
1.0
0.5
0.0
*
**
***
***
***
0
5
10
20
30
40
pmol of siRNA
B
ZR-75
KPNA1 mRNA expression level
1.5
1.0
0.5
0.0
*
**
***
***
***
0
5
10
20
30
40
pmol of siRNA
C
MCF-7
ZR-75
-
siCtrl
siKPNA1
-
siCtrl
siKPNA1
KPNA1
Actin

Figure S10

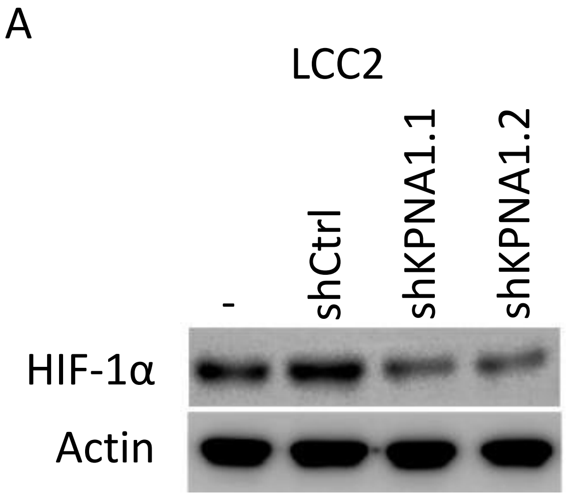
A
LCC2
-
shCtrl
shKPNA1.1
shKPNA1.2
HIF-1α
Actin

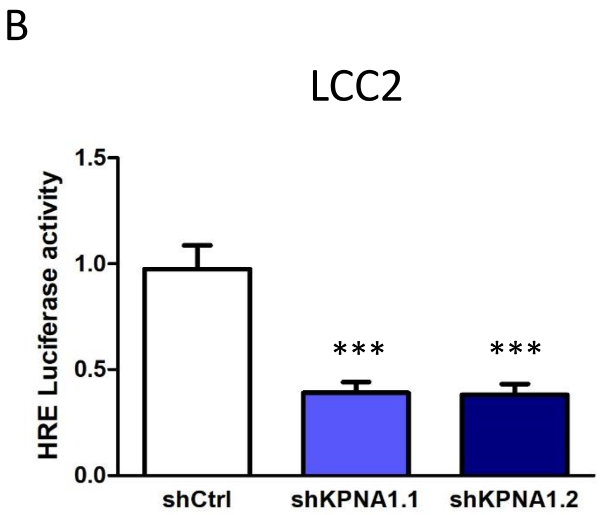
B
LCC2
HRE Luciferase activity
1.5
1.0
0.5
0.0
***
***
shCtrl
shKPNA1.1
shKPNA1.2

Figure S11

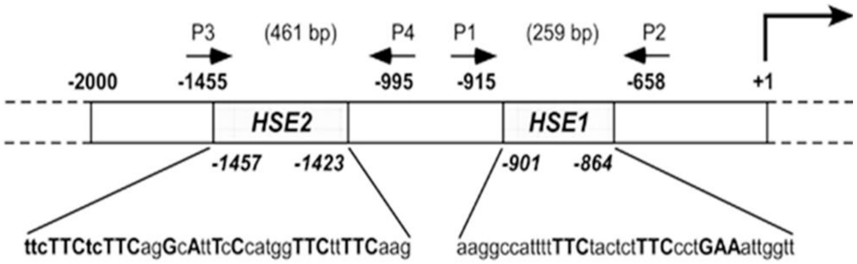

Adapted from Chen R, Liliental JE, Kowalski PE, Lu Q, Cohen SN. Regulation of transcription of hypoxia-inducible factor-1 alpha (HIF-1 alpha) by heat shock factors HSF2 and HSF4. Oncogene **2011**;30(22):2570-80 doi 10.1038/onc.2010.623.

Figure S12

Quantification of protein band intensity shown in Figure 1

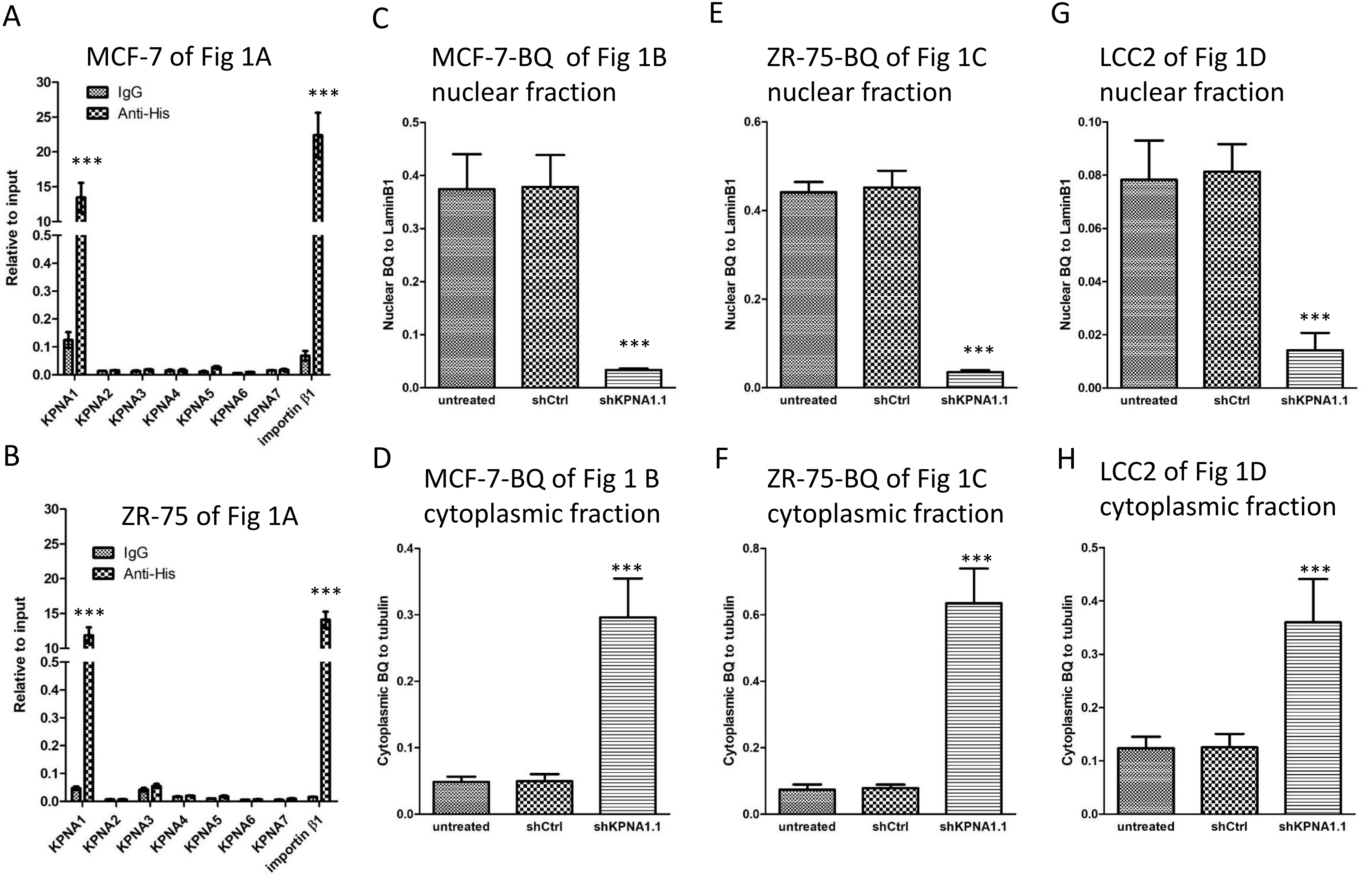

Figure S12 cont.

Quantification of protein band intensity shown in Figure 1

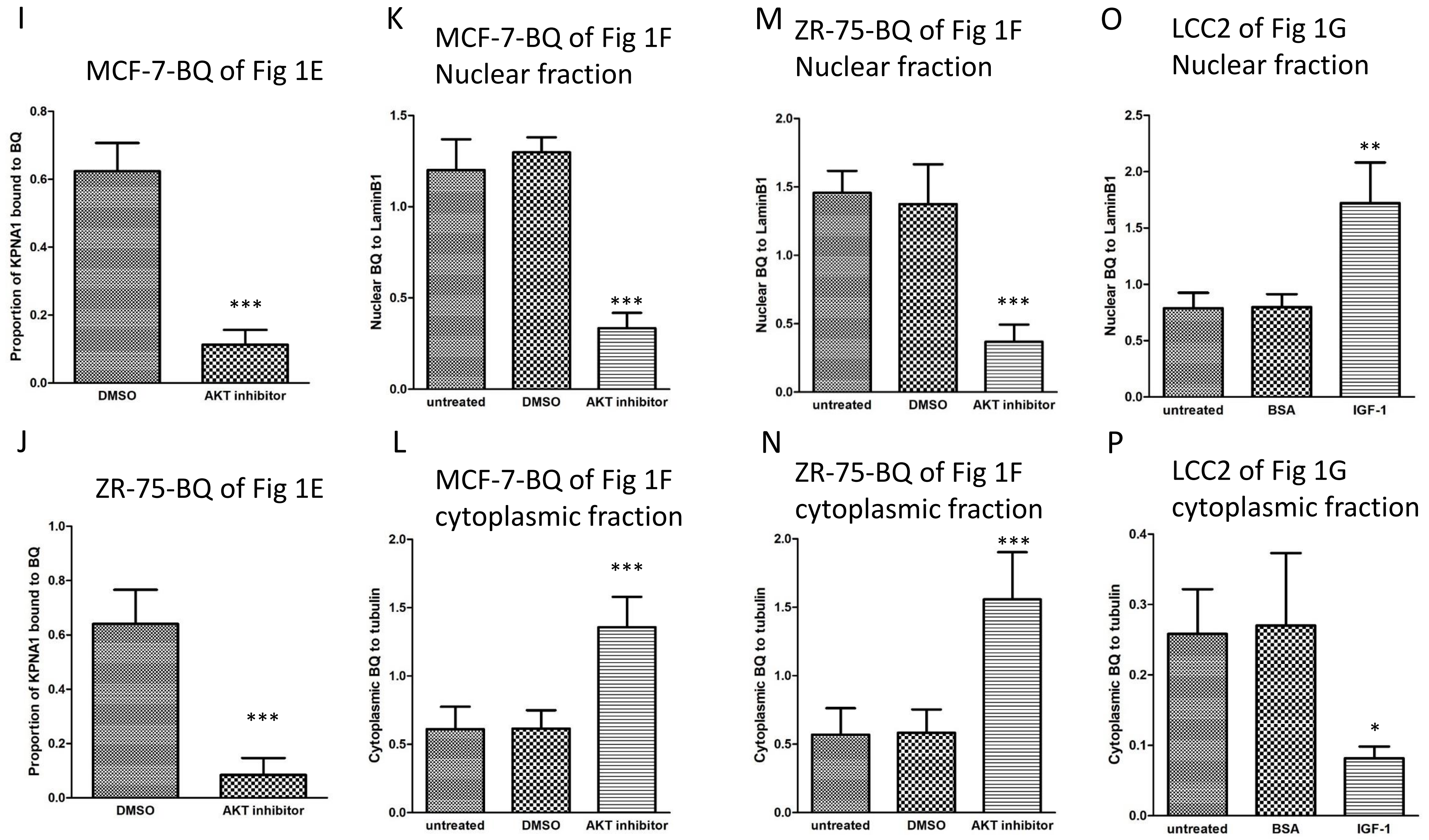

Figure S13

Quantification of protein band intensity shown in Figure 2

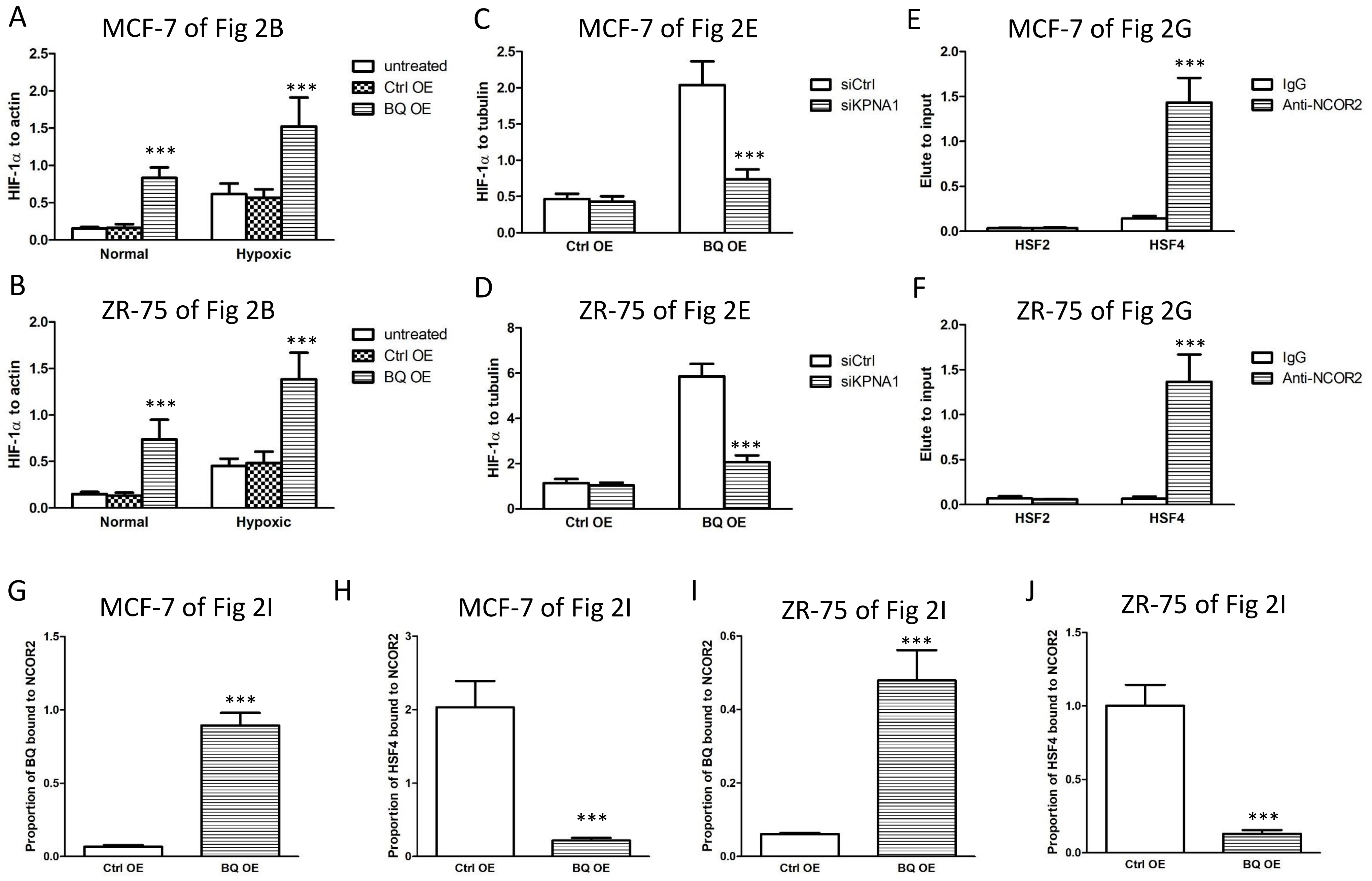

Figure S14 Quantification of protein band intensity shown in supplementary figures

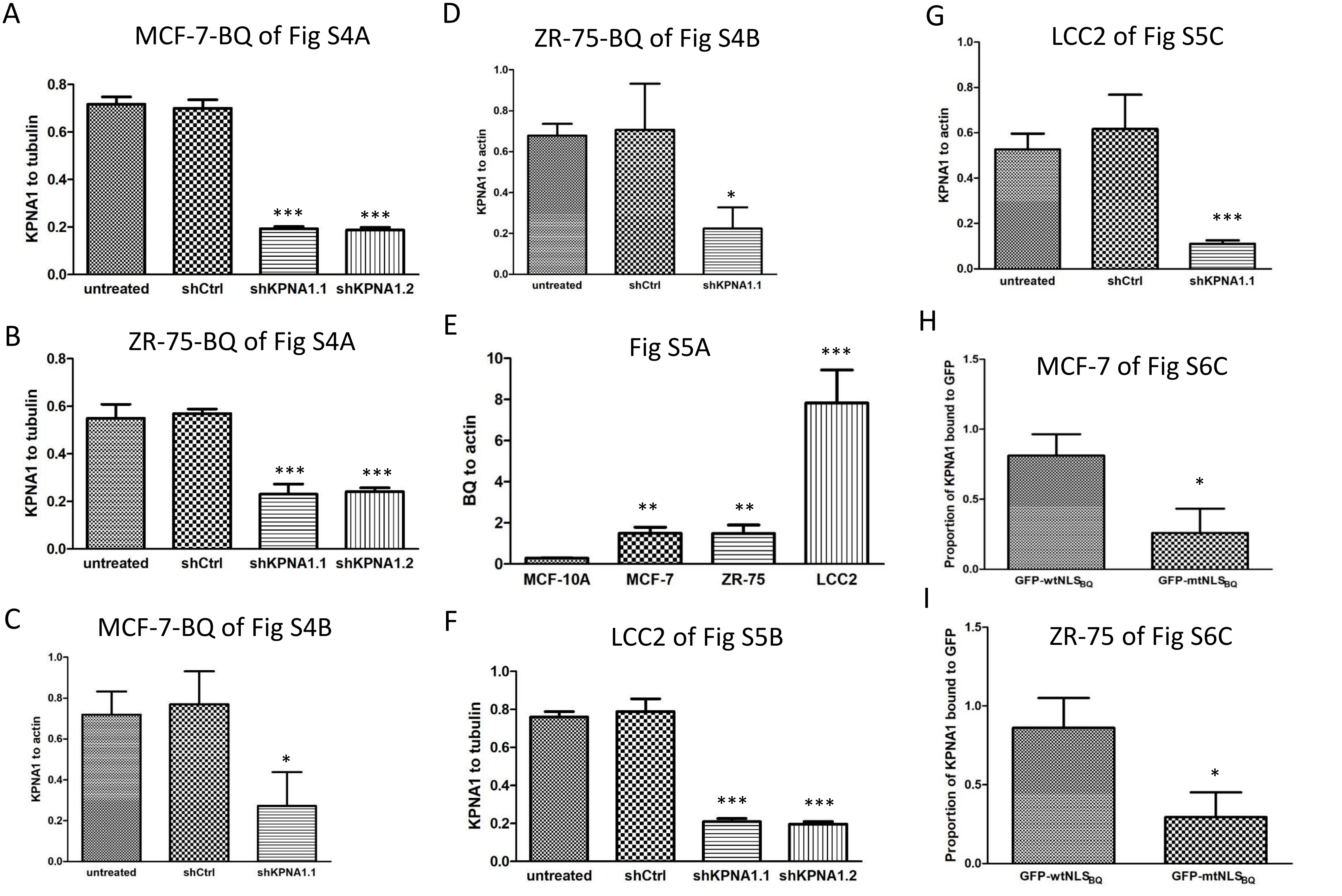

Figure S14 cont. Quantification of protein band intensity shown in supplementary figures

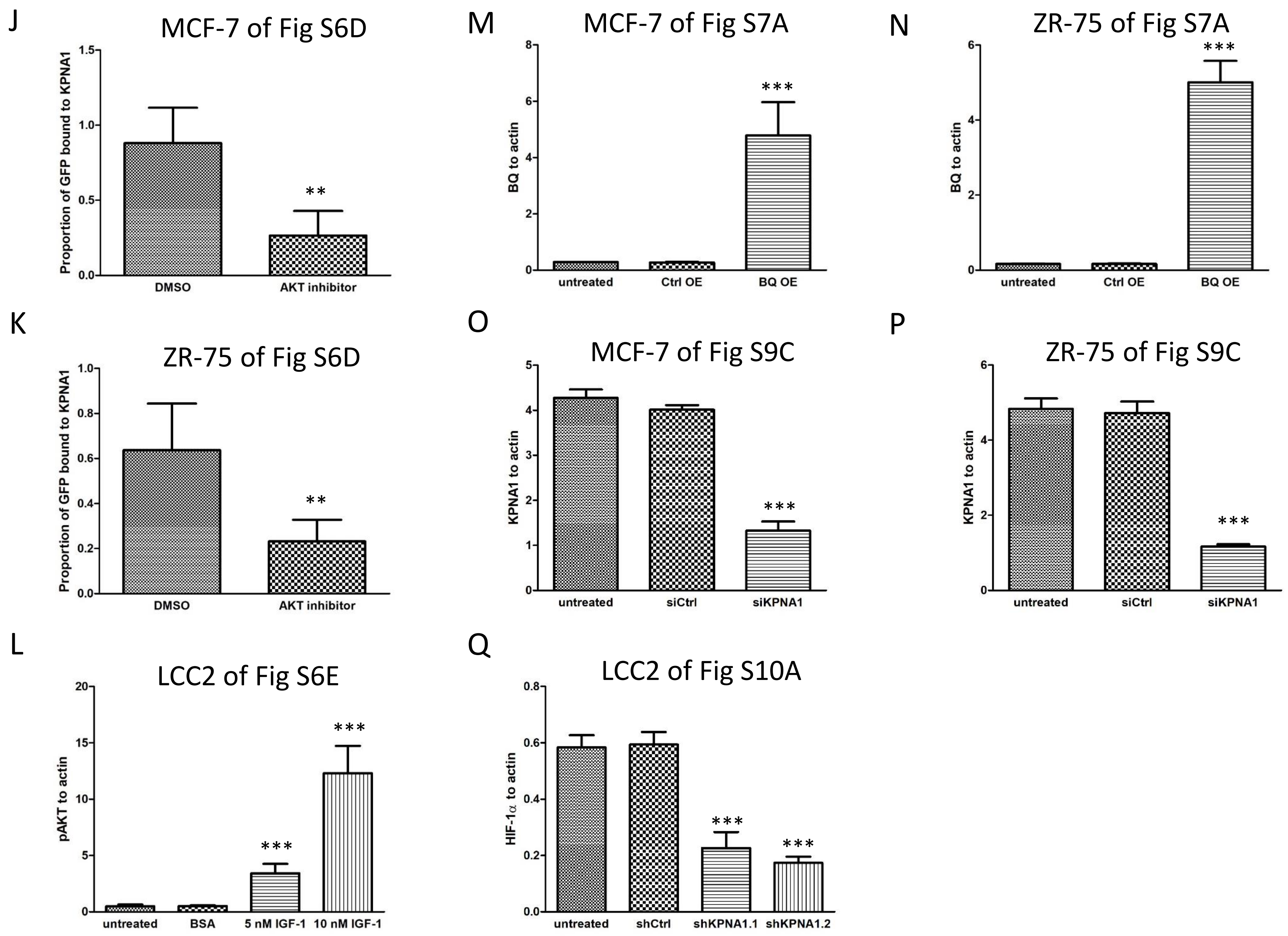

Supplement: Supplementary file 2 — Supporting Information [file CTM2-11-e554-s001.pdf]
