# Supplementary material for: KPNA1 regulates nuclear import of NCOR2 splice variant BQ323636.1 to confer tamoxifen resistance in breast cancer
Source: Clin Transl Med. 2021 Oct 12;11(10):e554. doi: 10.1002/ctm2.554 (PMC8506633; doi:10.1002/ctm2.554)
Supplement: Supplementary file 3 — Supporting Information [file CTM2-11-e554-s002.docx]

**Table S1 Clinico-pathologic characteristics of the breast cancer patients studied**

| **Clinico-pathologic characteristics** |  | **Number of Cases** | **Percentage (%)** |
| --- | --- | --- | --- |
| Breast cancer patients |  | 137 | 100 |
| Age | <56 | 65 | 47.4 |
|  | >=56 | 72 | 52.6 |
|  |  |  |  |
| T stage | I, II | 41 | 29.9 |
|  | III, IV | 10 | 7.2 |
|  | Missing | 86 | 62.9 |
|  |  |  |  |
| Lymph Node status | Positive | 67 | 48.9 |
|  | Negative | 56 | 40.9 |
|  | Missing | 14 | 10.2 |
|  |  |  |  |
| Tumor Grade | 1, 2 | 49 | 35.8 |
|  | 3 | 74 | 54.0 |
|  | Missing | 14 | 10.2 |
|  |  |  |  |
| Tumor Size | <2 cm | 37 | 27.0 |
|  | >=2 cm | 56 | 40.9 |
|  | Missing | 44 | 32.1 |
|  |  |  |  |
| Estrogen Receptor status | Positive | 71 | 51.8 |
|  | Negative | 25 | 18.3 |
|  | Missing | 41 | 29.9 |
|  |  |  |  |
| Progesterone receptor status | Positive | 49 | 35.8 |
|  | Negative | 37 | 27.0 |
|  | Missing | 51 | 37.2 |
|  |  |  |  |
| HER2 receptor status | Positive | 35 | 25.6 |
|  | Negative | 37 | 27.0 |
|  | Missing | 65 | 47.4 |
|  |  |  |  |
| Triple Negative status | Positive | 13 | 9.5 |
|  | Negative | 71 | 51.8 |
|  | Missing | 53 | 38.7 |
